# The “mechanical paradox” unveiled: a physiological study

**DOI:** 10.1186/s13054-025-05385-9

**Published:** 2025-05-16

**Authors:** Giorgia Pacchiarini, Tommaso Pettenuzzo, Francesco Zarantonello, Nicolò Sella, Gianluca Lumetti, Annalisa Boscolo, Alessandro De Cassai, Gianmaria Cammarota, Paolo Persona, Paolo Navalesi, the LOVE BEER Study Group, Giorgia Pacchiarini, Tommaso Pettenuzzo, Francesco Zarantonello, Nicolò Sella, Gianluca Lumetti, Annalisa Boscolo, Alessandro De Cassai, Gianmaria Cammarota, Paolo Persona, Paolo Navalesi, Francesco Monteleone, Gabriele Martelli, Ilaria Godi, Andrea Ballin, Ivo Tiberio, Arianna Peralta, Luisa Muraro, Enrico Petranzan, Elisa Pistollato

**Affiliations:** 1 Institute of Anesthesia and Intensive Care, University Hospital of Padua, 13 Via Gallucci, 35121 Padua, Italy; 2 Department of Medicine–DIMED, University of Padua, 2 Via Giustiniani, 35128 Padua, Italy; 3 Department of Cardiac, Thoracic, Vascular Sciences, and Public Health, University of Padua, Padua, Italy; 4 Department of Translational Medicine, Eastern Piedmont University, Novara, Italy; 5 Anesthesia and Intensive Care, University Hospital of Padua, Padua, Italy

**Keywords:** Respiration, Artificial [MeSH], Ventilator-induced lung injury [MeSH], Electrical impedance tomography, Esophageal pressure

## Abstract

**Background:**

Recent studies report that chest wall loading may reduce airway pressures and increase respiratory system compliance, contrary to the anticipated effect of this maneuver (“mechanical paradox”). Aim of this physiological study is to clarify the mechanism underlying this phenomenon.

**Methods:**

Twenty patients receiving invasive mechanical ventilation for acute hypoxemic respiratory failure were studied during a decremental PEEP trial. Variable weights were placed on the patients’ abdomen to achieve a 5-mmHg increase in intra-abdominal pressure. Three consecutive phases for each PEEP level were performed: weight-off, weight-on, and weight-off. Esophageal pressure measurement and electrical impedance tomography (EIT) were used.

**Results:**

The abdominal weight decreased end-expiratory lung impedance (EELI) and overdistention and increased collapse for all PEEP values (all *p*-values < 0.001). For PEEP values higher than the EIT-based optimal PEEP, the abdominal weight reduced respiratory system and lung plateau pressures (coefficient [standard error] − 1.26 [0.21] and − 5.51 [0.28], respectively, both *p*-values < 0.001) and driving pressures (− 1.47 [0.22] and − 1.62 [0.22], respectively, both *p*-values < 0.001). For PEEP values lower than the optimal, the effect of the application of the abdominal weight was the opposite (all *p*-values < 0.001).

**Conclusions:**

The improvement in respiratory system and lung mechanics following abdominal loading is consequent to the reduction of end-expiratory lung volume. This effect, however, only occurs at PEEP levels associated with prevalent overdistention. This simple and safe maneuver could be applied at the bedside to identify lung overdistension and titrate PEEP.

**Trial registration:**

ClinicalTrials.gov (NCT06174636, July 9th 2023).

**Supplementary Information:**

The online version contains supplementary material available at 10.1186/s13054-025-05385-9.

## Introduction

Positive end-expiratory pressure (PEEP) is currently employed as part of protective mechanical ventilation to minimize atelectrauma and improve oxygenation [1, 2]. Nonetheless, excessive PEEP may induce alveolar overdistension and hemodynamic compromise [3].

Recently, some studies, predominantly including patients with Coronavirus-19 disease (COVID-19) related acute respiratory distress syndrome (ARDS), found the application of a weight on the sternum or abdomen to reduce airway pressure (Paw), plateau pressure (Pplat), and driving pressure (DP) and to increase the quasi-static compliance (Cstat) of the respiratory system, contrary to the anticipated effect of this maneuver [4–12]. This phenomenon has been defined as “mechanical paradox” [4]. Some studies suggest the chest wall compression (rib cage or abdomen) as means to detect pulmonary overdistension, thus potentially allowing the titration of PEEP and tidal volumes (Vt) to reduce the risk of ventilator-induced lung injury [6, 8, 11, 12]. Marini et al. hypothesized that abdominal compression be preferable, not altering venous return compared to supradiaphragmatic compressions [13]. Most patients demonstrating the mechanical paradox were characterized by low respiratory system compliance, i.e., < 40 mL/cmH_2_O [6, 8, 12].

The underlying mechanisms of the mechanical paradox, however, are not fully elucidated yet. It was hypothesized it might depend on a reduction in end-tidal overdistention, with consequent improvement in lung compliance, as suggested by the leftward shift of the pressure–volume curve of the respiratory system following the application of the weight [13].

Aim of this physiological study is therefore to clarify the mechanisms underlying the mechanical paradox that occurs when positioning an abdominal weight in mechanically ventilated patients with acute hypoxemic respiratory failure (AHRF). For this purpose, we measured the effects of increasing intra-abdominal pressure (IAP) by 5 mmHg, at varying PEEP levels, on the mechanical properties of lung, chest wall, and overall respiratory system and on the end-expiratory lung impedance (EELI), collapse (CL) and overdistension (OD), as assessed by electrical impedance tomography (EIT). Furthermore, hypothesizing the effect of weight application to depend on whether PEEP exceeded optimal PEEP (overdistension prevailing) or not (collapse prevailing), we also aimed to confirm under which conditions the maneuver may help identify the occurrence of overdistension.

## Methods

### Study design

This prospective interventional single-center study, approved by the Ethics Committee for Clinical Trials of the Province of Padua (protocol 5756/AO/23) and registered on ClinicalTrials.gov (NCT06174636, July 9th 2023), was conducted in accordance with the principles of the Helsinki Declaration. Informed consent was obtained according to national regulation. We enrolled all consecutive patients conforming to the following inclusion criteria: (1) age ≥ 18 years old; (2) invasive mechanical ventilation for AHRF (arterial partial pressure of oxygen [PaO2] to fraction of inspired oxygen [FiO2] ratio < 300 mmHg). The exclusion criteria were: (1) contraindications to the use of EIT, e.g., pace-makers or devices with metal components, burns, surgical dressings at the thoracic level [14]; (2) contraindications to the positioning of an abdominal weight, e.g., surgical incisions, recent, i.e., < 14 days, abdominal wounds, severe abdominal hypertension, i.e., baseline IAP > 20 mmHg; (3) contraindications to the positioning of the esophageal catheter, i.e., esophageal diseases, such as ulcerations, tumors, diverticulitis, bleeding varices, or sinusitis, epistaxis or recent nasopharyngeal surgery [15]; (4) severe hemodynamic instability, i.e., use of norepinephrine or epinephrine > 0.1 μg/kg/min, dobutamine or dopamine > 5 μg/kg/min; (5) class II obesity, i.e., body mass index (BMI) ≥ 35 kg/m2; (6) pregnancy; (7) no informed consent.

### Study protocol

Before starting the protocol, the esophageal balloon, the EIT belt, and the urinary catheter (if not already in place) were positioned. All patients were studied in the semi-recumbent position while deeply sedated (i.e., Richmond Agitation and Sedation Scale = − 5) and paralyzed with continuous infusion of cisatracurium to achieve a train-of-four score of 0–1. In cases where the dose of neuromuscular blocker was not adequate, an additional 0.15 mg/kg dose of rocuronium bromide was administered. Mechanical ventilation was applied in flow-limited volume-cycled controlled mode with tidal volume ≤ 6 mL/kg of predicted body weight (PBW) and driving pressure ≤ 14 cmH_2_O, while PEEP was initially set according to clinical indications. Respiratory rate (RR) was regulated to achieve zero flow at the end of expiration, while maintaining pH > 7.30. FiO_2_ was kept stable throughout the study.

The study protocol involved the following steps:

1. Start of the EIT registration;
2. Recruitment maneuver with continuous positive airway pressure (CPAP) at 30 cmH_2_O for 30 s;
3. Setting of PEEP at 20 cmH_2_O;
4. After 10 breaths, a 3-s end-inspiratory hold, followed by a 3-s end-expiratory hold in the following breath;
5. Application of a weight on the epi-mesogastric region, consisting of bags of normal saline solution, to achieve an increase in IAP of 5 mmHg, compared to the baseline value;
6. After 10 breaths, a 3-s end-inspiratory hold, followed by a 3-s end-expiratory hold in the following breath;
7. Weight removal;
8. After 10 breaths, a 3-s end-inspiratory hold, followed by a 3-s end-expiratory hold in the following breath;
9. Recruitment maneuver with CPAP at 30 cmH_2_O for 30 s;
10. Reduction of the PEEP level by 2 cmH_2_O.

All steps from 4 to 9 were repeated until a PEEP level of 8 cmH_2_O was reached, with this last level included. Overall, the protocol involved 7 levels of PEEP and, for each PEEP level, three phases: phase 1 (weight-off); phase 2 (weight-on); phase 3 (weight-off).

### Intra-abdominal pressure measurement

A pressure transducer was connected to the bladder catheter and positioned at the iliac crest level along the mid-axillary line. The urinary catheter was clamped distal to the transducer and 25 mL of normal saline solution were injected into the bladder. The transducer was connected to the multiparametric monitor to display the IAP value and curve continuously throughout the study [16].

### Esophageal pressure measurement

The Cooper Surgical (Trumbull, Connecticut, USA) adult esophageal balloon catheter set was used. After checking its integrity and connecting it to the PulmoVista 500 (Dräger Lübeck, Germany) monitoring system, lidocaine 2% spray was applied to the patient’s nasal passage and throat and the catheter was inserted through the nasopharynx until the middle-inferior portion of the intrathoracic esophagus. The catheter final depth was estimated by calculating the product of the patient’s height (cm) × 0.288.

After evacuating all the air, the balloon was inflated at 1-mL steps with air. The optimal inflation volume was identified as the minimum volume leading to the greatest difference between peak-inspiratory and end-expiratory esophageal pressure (Pes). The Baydur maneuver was then performed, consisting of two compressions on the patient's sternum during an end-expiratory hold to verify that the ratio between the changes in Pes and Paw was between 0.8 and 1.2 [15]. We repeated the calibration maneuver whenever the curve quality deteriorated. Once the balloon was inserted and calibrated, the transpulmonary pressure (Pl) was calculated as the difference between Paw and Pes [15].

### Electrical impedance tomography

An adequately sized EIT belt was positioned at the fifth intercostal space and connected to the PulmoVista 500 (Dräger Lübeck, Germany) device. In case of reduced signal quality, normal saline solution was applied under the individual electrodes to improve their signal.

After calibration, the tidal impedance variation (TIV) in the whole lung and in four regions of interest, i.e., ventral, mid-ventral, mid-dorsal, and dorsal, was assessed. The analysis was performed offline with the Pulmovista EIT Diag software (version 1.6). The curves representing the cumulative percentage of compliance loss due to either collapse or overdistension were obtained and optimal PEEP was considered as the level corresponding to the intersection between these two curves [17]. The percent of overdistension was higher than the percent of collapse for PEEP levels higher than the optimal; for lower levels, the percent of collapse was higher than that of overdistension.

### Data collection

Before starting the protocol, patients’ age, gender, weight, height, BMI, PBW, and the underlying etiology of AHRF were collected. Moreover, ventilator settings (Vt, RR, and PEEP), and arterial blood gas exchanges (pH, arterial partial pressure of carbon dioxide, PaO_2_/FiO_2_) were recorded. Once the protocol was started, the following outcome variables were recorded for all PEEP levels in each study phase, i.e., phase 1, phase 2, and phase 3:

- Respiratory mechanics related to the respiratory system (RS), the lung (L) and the chest wall (CW), i.e., plateau pressure (Pplat_RS, Pplat_L, Pplat_CW), driving pressure (DP_RS, DP_L, DP_CW), and quasi-static compliance (Cstat_RS, Cstat_L, Cstat_CW); peak pressure was collected for the respiratory system only (Ppeak_RS).
- EIT derived variables: the percentages of EIT-derived OD and collapse CL; the EELI, compared to the value at 8 cmH_2_O of PEEP during phase 2 (ΔEELI), estimating the variation in end-expiratory lung volume with respect to the phase with greater expected collapse; the global inhomogeneity (GI) index, estimating the inhomogeneity of tidal volume distribution [14]. Each of these variables was evaluated both in the whole lung and in individual lung regions, i.e. the ventral, mid-ventral, mid-dorsal, and dorsal areas.

### Statistical analysis

Quantitative data are presented as median and 1^st^–3^rd^ quartile, while qualitative data as number and percentage. The clinical and anamnestic variables, IAP values, and values of the weights used during the study are reported as individual values for each patient. We aimed to enroll 20 patients.

The effects, on each outcome variable, of the two interventions, i.e., the abdominal weight application and PEEP, were evaluated. PEEP was not considered as absolute value, but as the difference (ΔPEEP) between the set PEEP level and the EIT-based optimal PEEP, as defined above, identified during phase 1. We hypothesized that the effect of PEEP could depend on whether PEEP was higher (i.e., overdistension prevailing on collapse) or lower (i.e., collapse overcoming overdistension) than optimal PEEP. Therefore, the effect of ΔPEEP on outcome variables was analyzed separately for positive and negative values, respectively. For pairwise comparisons relative to the effect of PEEP, ΔPEEP of 0 cmH_2_O was considered as reference for positive ΔPEEP values, while ΔPEEP of − 8 cmH_2_O was considered as reference for negative ΔPEEP values. For pairwise comparisons relative to the effect of weight application, phase 1 was considered as reference.

Linear mixed-effects models were used to evaluate the association between each outcome variable and the two interventions. The model included the individual patient as a random factor and the effect of weight and ΔPEEP as fixed factors. The estimates, standard errors, and p-values of the association are reported. Linear mixed-effects models were conducted for each ΔPEEP level and study phase for pairwise comparisons relative to the effect of weight application and PEEP, respectively.

All statistical tests were two-tailed and statistical significance was defined by a *p* < 0.05. Analyses were conducted using R (version 4.3.3, R Foundation for Statistical Computing, Vienna, Austria).

## Results

From June 2023 to March 2024, 20 patients with AHRF were enrolled. Patients’ characteristics and ventilator settings on study entry are reported in Table 1 and Table E1 (Additional File 1), respectively. Median age was 67 (51–75) years old and two patients (10%) were female. Sixteen patients (80%) suffered from pneumonia. Tidal volume was 5.8 (5.1–6.2) mL/kg of predicted body weight and PEEP was 14 (10–14) cmH_2_O. Baseline Cstat_RS was 52 (40–65) mL/cmH_2_O. Table E2 (Additional File 1) shows the variations in IAP at the various study phases and at different PEEP levels. The abdominal weight necessary to obtain a 5-mmHg increase in IAP ranged between 5 and 11 kg. No complication related to the application of the abdominal weight was observed.

**Table 1:** Baseline characteristics of the patients

Patient	AHRF etiology	Age (years)	Gender	Height (m)	Weight (kg)	PBW (kg)	BMI (kg/m^2^)
1	Pneumonia with ARDS	77	F	1.65	70	57	26
2	Pneumonia with ARDS	54	M	1.80	100	75	31
3	Trauma with ARDS	43	M	1.80	110	75	34
4	Post-operative AHRF	77	F	1.60	70	52	27
5	Pneumonia with ARDS	72	M	1.70	70	66	24
6	Pneumonia	84	M	1.70	80	66	28
7	Legionella pneumonia	51	M	1.85	78	80	23
8	Pneumonia with ARDS	68	M	1.70	75	66	26
9	Severe acute asthma	33	M	1.75	75	71	25
10	Drug toxicity	36	M	1.80	95	75	29
11	Pneumonia with ARDS	76	M	1.80	100	75	31
12	Pneumonia	66	M	1.60	75	59	29
13	Septic shock	68	M	1.70	100	67	35
14	Septic shock	80	M	1.75	90	70	29
15	H1N1 pneumonia	74	M	1.82	90	76	27
16	Legionella pneumonia	68	M	1.80	85	75	26
17	Pneumonia after ROSC	64	M	1.70	100	67	35
18	H1N1 pneumonia	53	M	1.80	96	75	30
19	Post-operative pneumonia	47	M	1.75	105	70	34
20	H. influenzae pneumonia	51	M	1.80	110	75	34

*AHRF* acute hypoxemic respiratory failure, *PBW* predicted body weight, *BMI* body mass index, *ARDS* acute respiratory distress syndrome, *ROSC* return of spontaneous circulation

### Respiratory mechanics

**Fig. 1:**
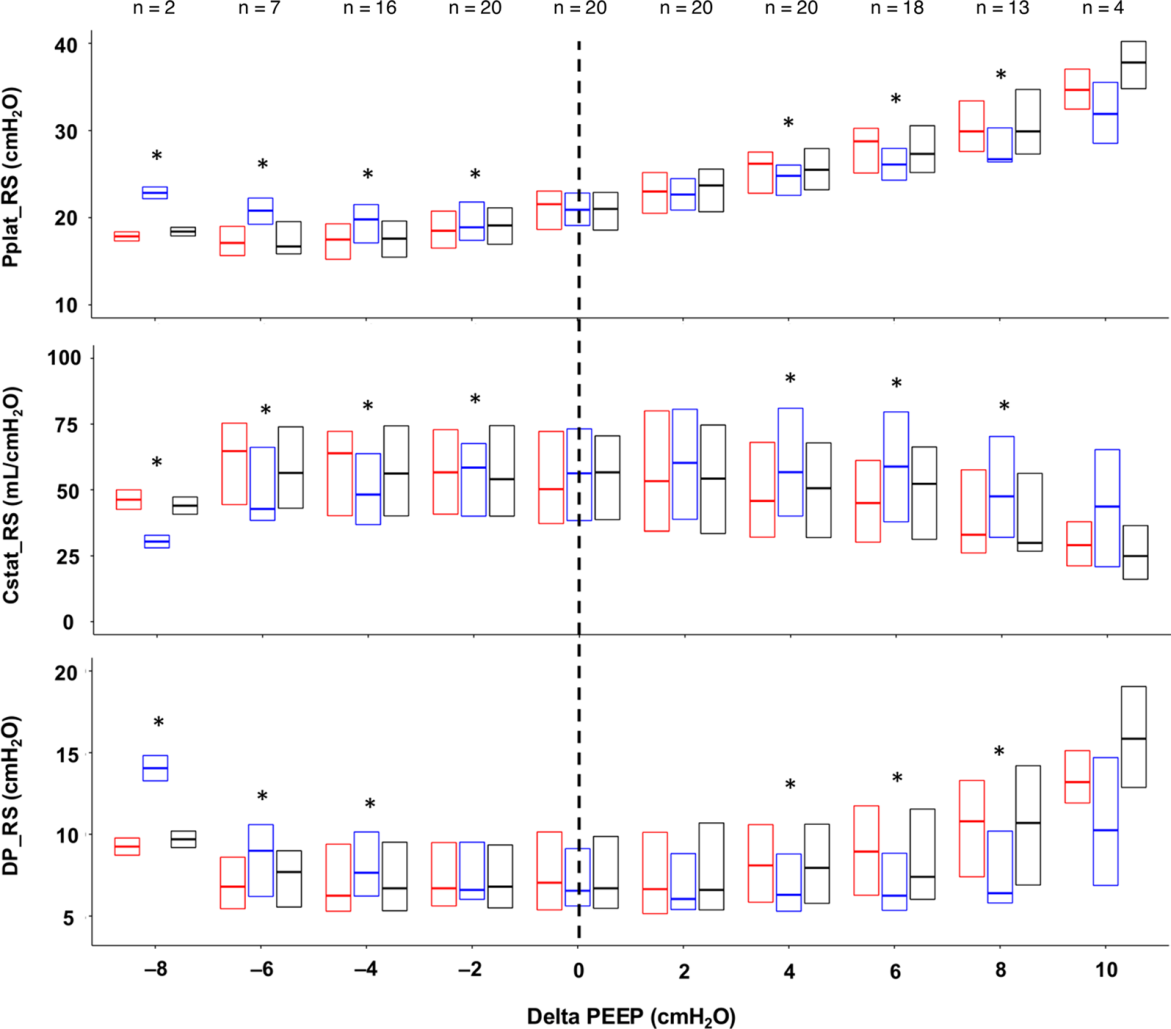
Variation of Pplat_RS, Cstat_RS, and DP_RS with the different study phases and ΔPEEP. Each boxplot shows the median, 1st, and 3rd quartile of the variable value. Phase 1, phase 2, and phase 3 are depicted in red, blue, and black, respectively. Not all variables could be measured for each ΔPEEP level because of the fixed PEEP range explored in the decremental PEEP trial and the varying best PEEP value for each patient. The number of patients with available ΔPEEP values is displayed in the figure. *Abbreviations*: PEEP, positive end-expiratory pressure; Pplat, plateau pressure; RS, respiratory system; Cstat, static compliance; DP, driving pressure. The dotted vertical line identifies the ΔPEEP 0 cmH_2_O value. *p < 0.05 of the pairwise comparison between phase 2 vs. phase 1 within each PEEP level

**Fig. 2:**
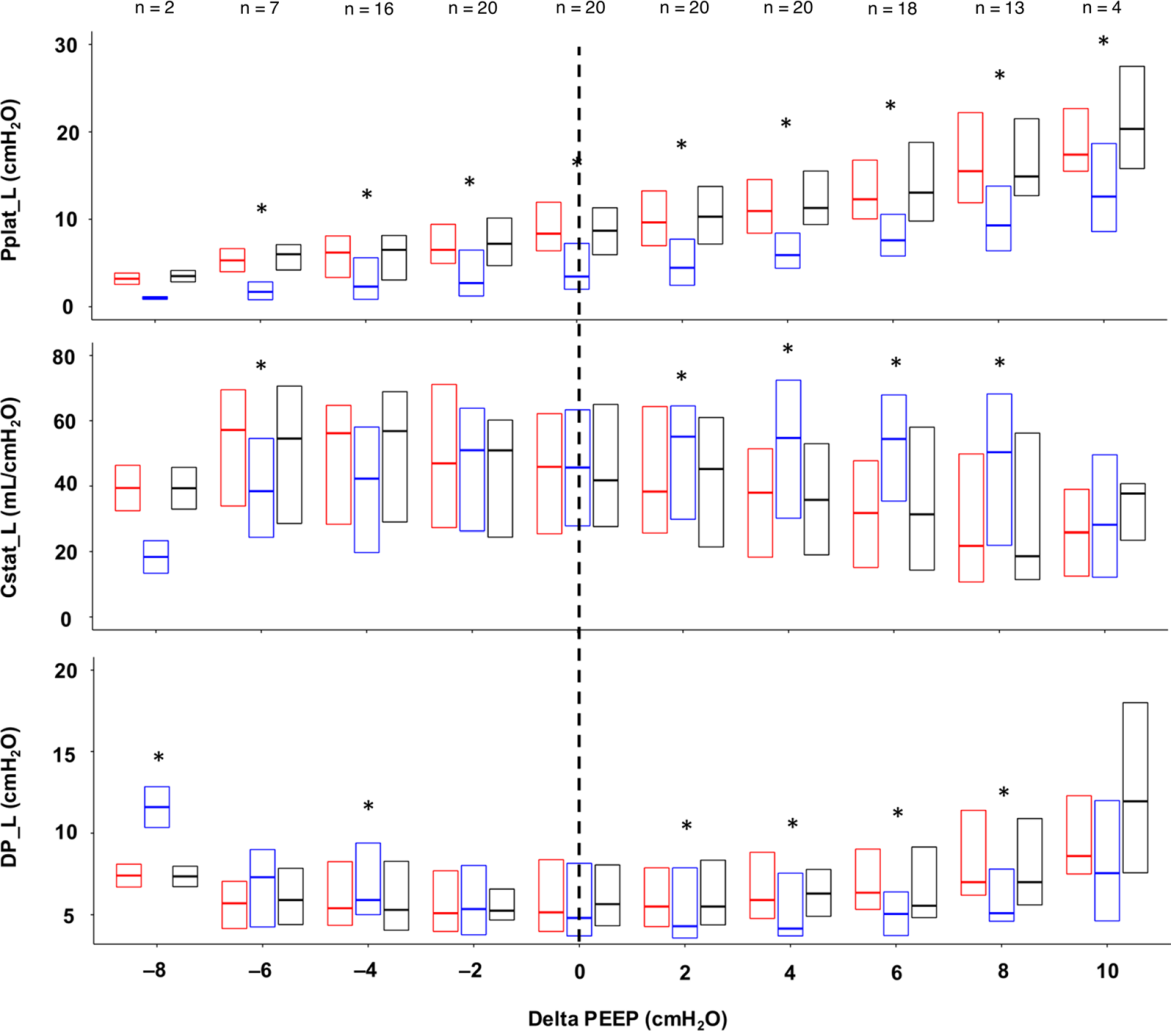
Variation of Pplat_L, Cstat_L, and DP_L with the different study phases and ΔPEEP. Each boxplot shows the median, 1st, and 3rd quartile of the variable value. Phase 1, phase 2, and phase 3 are depicted in red, blue, and black, respectively. Not all variables could be measured for each ΔPEEP level because of the fixed PEEP range explored in the decremental PEEP trial and the varying best PEEP value for each patient. The number of patients with available ΔPEEP values is displayed in the figure. *Abbreviations*: PEEP, positive end-expiratory pressure; Pplat, plateau pressure; L, lung; Cstat, static compliance; DP, driving pressure. The dotted vertical line identifies the ΔPEEP 0 cmH_2_O value. *p < 0.05 of the pairwise comparison between phase 2 vs. phase 1 within each PEEP level

Figure 1 depicts the variation of Pplat_RS, Cstat_RS, and DP_RS, while Fig. 2 the variation of Pplat_L, Cstat_L, and DP_L, following the application of the two interventions. The linear mixed-effects models relative to the effect of weight application are shown in Table 2. Table E3 and Figure E1 (Additional File 1) display the changes in delta-PEEP 0 across the three study phases for each patient. The complete report of lung, chest wall, and respiratory system mechanics is presented in Table E4 (Additional File 1), while the effects of the different levels of ΔPEEP on respiratory mechanics variables are reported in Table E5 (Additional File 1).

**Table 2:** Effect of abdominal loading on respiratory mechanics

Variable	PEEP level	Coefficient	Standard error	*p*-value
Ppeak_RS (cmH_2_O)	≥ best PEEP	− 1.21	0.27	< 0.001
	< best PEEP	3.33	0.29	< 0.001
Pplat_RS (cmH_2_O)	≥ best PEEP	− 1.26	0.21	< 0.001
	< best PEEP	2.09	0.20	< 0.001
Cstat_RS (mL x cmH_2_O^−1^)	≥ best PEEP	7.36	1.10	< 0.001
	< best PEEP	− 7.93	1.39	< 0.001
Pplat_L (cmH_2_O)	≥ best PEEP	− 5.51	0.28	< 0.001
	< best PEEP	− 3.35	0.28	< 0.001
Cstat_L (mL x cmH_2_O^−1^)	≥ best PEEP	22.03	3.22	< 0.001
	< best PEEP	− 6.35	3.45	0.069

The coefficients, standard errors, and p-values of the association between ΔPEEP, i.e., the difference between the set PEEP level and the EIT-based optimal PEEP, and each outcome variable are obtained through the application of mixed-effects models, including the individual patient as a random factor and the weight and ΔPEEP as fixed factors

*PEEP* positive end-expiratory pressure, *Ppeak* peak pressure, *RS* respiratory system, *Pplat* plateau pressure, *Cstat* static compliance, *L* lung

For PEEP values higher than the optimal PEEP, the application of the abdominal weight (phase 2 vs. phase 1) was associated with a significant reduction in Ppeak_RS (coefficient − 1.21; SE 0.27), Pplat_RS (coefficient − 1.26; SE 0.21), DP_RS (coefficient − 1.47; SE 0.22), Pplat_L (coefficient − 5.51; SE 0.28), PEEP_L (coefficient − 3.89; SE 0.18), and DP_L (coefficient − 1.62; SE 0.22) (all *p*-values < 0.001) and a significant increase in Cstat_RS (coefficient 7.36; SE 1.1), Cstat_L (coefficient 22.03; SE 3.22), and Pplat_CW (coefficient 4.25; SE 0.20) (all *p*-values < 0.001). Cstat_CW decreased during phase 2, though not significantly (coefficient − 15.13; SE 77.91; *p*-value 0.846).

For PEEP values lower than the optimal PEEP, the application of the abdominal weight was associated with a significant increase in the values of Ppeak_RS (coefficient 3.33; SE 0.29), Pplat_RS (coefficient 2.09; SE 0.20), DP_RS (coefficient 1.12; SE 0.21), DP_L (coefficient 0.69; SE 0.20), and Pplat_CW (coefficient 5.48; SE 0.29) (all *p*-values < 0.001) and a significant reduction in Cstat_RS (coefficient − 7.93; SE 1.39), Pplat_L (coefficient − 3.35; SE 0.28), and PEEP_L (coefficient − 4.05; SE 0.28) (all *p*-values < 0.001). Cstat_CW increased during phase 2, though not significantly (coefficient 78.42; SE 158.24; *p*-value 0.621).

Pairwise comparisons between different ΔPEEP levels and study phases are reported in Table E6 (Additional File 1).

### Electrical impedance tomography

**Fig. 3:**
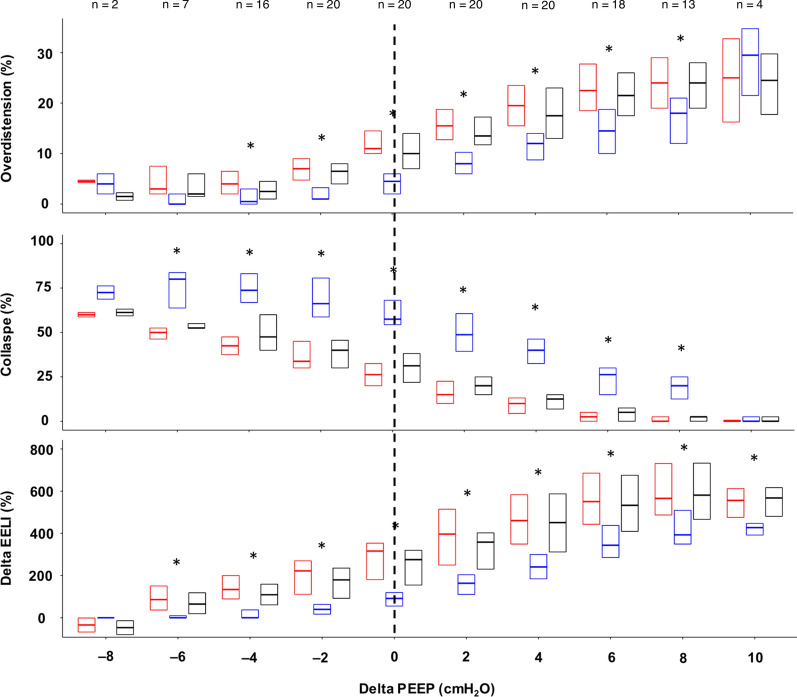
Variation of overdistention, collapse, and ΔEELI with the different study phases and ΔPEEP. Each boxplot shows the median, 1st, and 3rd quartile of the variable value. Phase 1, phase 2, and phase 3 are depicted in red, blue, and black, respectively. Not all variables could be measured for each ΔPEEP level because of the fixed PEEP range explored in the decremental PEEP trial and the varying best PEEP value for each patient. The number of patients with available ΔPEEP values is displayed in the figure. *Abbreviations*: PEEP, positive end-expiratory pressure; ΔEELI, difference of end-expiratory lung impedance compared to the value at 8 cmH_2_O of PEEP during phase 2. The dotted vertical line identifies the ΔPEEP 0 cmH_2_O value. *p < 0.05 of the pairwise comparison between phase 2 vs. phase 1 within each PEEP level

Figure 3 depicts the variation of OD, CL, and ΔEELI following the application of the two interventions. The linear mixed-effects models relative to the effect of weight application are shown in Table 3. The complete report of EIT variables is present in Table E7 (Additional File 2), while the effects of the different levels of ΔPEEP on EIT variables is reported in Table E8 (Additional File 2).

**Table 3:** Effect of abdominal loading on EIT variables

Variable	PEEP level	Coefficient	Standard error	*p*-value
OD_tot (%)	≥ best PEEP	− 7.18	0.49	< 0.001
	< best PEEP	− 4.58	0.63	< 0.001
CL_tot (%)	≥ best PEEP	10.64	0.65	< 0.001
	< best PEEP	11.51	0.87	< 0.001
ΔEELI_tot (%)	≥ best PEEP	− 202.51	9.81	< 0.001
	< best PEEP	− 133.18	13.09	< 0.001

The coefficients, standard errors, and p-values of the association between ΔPEEP, i.e., the difference between the set PEEP level and the EIT-based optimal PEEP, and each outcome variable are obtained through the application of mixed-effects models, including the individual patient as a random factor and the weight and ΔPEEP as fixed factors

*PEEP* positive end-expiratory pressure, OD, lung overdistension; tot, total, i.e., referred to the entire lung; CL, lung collapse; ΔEELI, difference of end-expiratory lung impedance compared to the value at 8 cmH_2_O of PEEP during phase 2

For PEEP values higher than the optimal PEEP, the application of the abdominal weight (phase 2 vs. phase 1) was associated with a significant decrease in OD (coefficient − 7.18, SE 0.49), global ΔEELI (coefficient − 202.51; SE 9.81), dorsal ΔEELI (coefficient − 30.42; SE 1.94), mid-dorsal ΔEELI (coefficient − 72.24; SE 3.80), mid-ventral ΔEELI (coefficient − 68.32; SE 5.52), and ventral ΔEELI (coefficient − 31.17; SE 2.34) and a significant increase in CL (coefficient 10.64; SE 0.65), and GI (coefficient 8.22; SE 0.85) (all *p*-values < 0.001). The removal of the weight (phase 3 vs. phase 1) was also associated with significantly decreased OD (coefficient − 1.30; SE 0.49; *p*-value 0.008).

For PEEP levels lower than the optimal, the application of the abdominal weight was associated with a significant decrease in OD (coefficient − 4.58; SE 0.63), global ΔEELI (coefficient − 133.18; SE 13.09), dorsal ΔEELI (coefficient − 14.38; SE 1.84), mid-dorsal ΔEELI (coefficient − 37.44; SE 4.11), mid-ventral ΔEELI (coefficient − 50.56; SE 6.88), and ventral ΔEELI (coefficient − 30.64; SE 3.34) (all *p*-values < 0.001), and a significant increase in CL (coefficient 11.51; SE 0.87) and GI (coefficient 14.60; SE 1.47) (both *p*-values < 0.001).

Pairwise comparisons between different ΔPEEP levels and study phases are reported in Table E9 (Additional File 2).

## Discussion

In our cohort of patients receiving controlled mechanical ventilation for AHRF, we found significantly different effects produced by the abdominal loading depending on the prevalence of lung overdistension or collapse. In the case of prevailing PEEP-related lung overdistension, i.e., above the optimal PEEP as assessed by EIT [17], the abdominal weight reduced driving pressure and increased quasi-static compliance of the lung and respiratory system. In contrast, in the case of prevailing PEEP-related lung collapse, i.e., below the optimal PEEP, weight application increased driving pressure while decreasing lung and respiratory system quasi-static compliance. Irrespective of the PEEP level, the abdominal load decreased overdistension and increased collapse, reducing both global and regional EELI, a surrogate of the end-expiratory lung volume. While confirming the hypothesis that the reduction in end-expiratory lung volume might have a role in explaining the mechanical paradox by avoiding or reducing overdistension [13], we further elucidate the mechanism showing that the reduction in EELI improves respiratory mechanics only when overdistension prevails on collapse. Noteworthy, the reduction in EELI consequent to abdominal loading was observed mainly in the mid-ventral and mid-dorsal lung regions, indicating that the lower overdistension depends on the loading effect primarily on these regions.

Our results are in keeping with the recent study by Moncomble et al. [11], who compressed the rib cage of 20 patients with ARDS by means of a weight imposed on the sternum at two PEEP levels, and observed a decrease in airway Pplat at the higher PEEP, determining prevalent overdistention, and an increase in airway Pplat at the lower PEEP, associated with prevalent collapse. Worth remarking, however, different from that study, we consider 7 PEEP levels and also partition respiratory mechanics in the lung and chest wall components.

In COVID-19 patients with low respiratory system compliance showing a paradoxical response to rib cage loading, Selickman et al. [6] observed improved respiratory system compliance following Vt and/or PEEP reduction, which they attributed to the presence of lung overdistension. In the same patient population, Bastia et al. [12] and Umbrello et al. [8] also found rib cage loading to increase respiratory system compliance. These two studies also concluded that reduced overdistention may be the main determinant of this phenomenon, though only in the patients with lower baseline compliance. In keeping with these studies, we found abdominal loading to improve respiratory system compliance; however, by performing a decremental PEEP trial, we observed that the effect of abdominal loading, irrespective of baseline respiratory system compliance, depended on the amount of PEEP, with the improvement in lung and respiratory mechanics occurring only for PEEP values determining prevailing overdistension.

Our results indicate that abdominal loading may help to identify lung overdistension and guide PEEP selection: when peak, plateau, and driving pressure of the respiratory system decrease following weight application, the applied PEEP exceeds the optimal level and should be reduced.

Applying an abdominal weight is simple, safe, and does not require advanced monitoring. Anyhow, several questions still remain unaddressed. Different from most previous investigations [5–12], we placed the weight on the abdomen rather than on the rib cage in order to reduce the effect of loading on venous return, as advocated for by Marini and Gattinoni [13]. Furthermore, the application of the weight on the rib cage produces a vertical vector on the chest wall, while abdominal loading is likely to generate a lateral vector secondary to the cephalad displacement of the diaphragm [18]. Nonetheless, whether the best site for chest wall loading is the sternum or the abdomen remains to be clarified. Other issues deserve further investigation, such as the efficacy of abdominal loading in titrating tidal volume, the ideal pressure to be applied to unveil the mechanical paradox, and the effects in severely obese patients.

This study has a few points of strengths. Different from all previous studies, we titrated the weight application to a predetermined increase in IAP of 5 mmHg, which was obtained with a quite broad range of applied weights (between 5 and 11 kg). Also, we did not limit our investigation to COVID-19 patients or ARDS patients with reduced compliance of the respiratory system, thus adding to the generalizability of our results. Finally, our study suggests a possible clinical application that deserves, nonetheless, further investigation. There are also limitations. First, the sample size was not determined because of the lack of solid data allowing its calculation, a limit that we share with several physiological studies. Second, the single-center design, which is justified by the complexity of the protocol and the need for strict control of its application. Third, the choice of the method to determine the optimal PEEP is discretional, though commonly applied, and other strategies based on quasi-static respiratory system compliance, pressure–volume curves, or other EIT indices could have been used [14].

In conclusion, our study shows that the improvement in respiratory system and lung compliance following abdominal loading in mechanically ventilated patients, the so-called “mechanical paradox”, is consequent to the reduction of end-expiratory lung volume. This effect, however, only occurs at PEEP levels associated with prevalent overdistention. Whether this simple and safe maneuver could be applied at the bedside to titrate PEEP requires further investigation with a multi-center study.

## Supplementary Information

Additional file 1.

Additional file 2.

## Data Availability

No datasets were generated or analysed during the current study.

## Abbreviations

PEEP: Positive end-expiratory pressure
COVID-19: Coronavirus-19 disease
ARDS: Acute respiratory distress syndrome
Paw: Airway pressure
Pplat: Plateau pressure
DP: Driving pressure
Cstat: Quasi-static compliance
Vt: Tidal volumes
AHRF: Acute hypoxemic respiratory failure
IAP: Intra-abdominal pressure
EELI: End-expiratory lung impedance
CL: Collapse
OD: Overdistension
EIT: Electrical impedance tomography
PaO2: Arterial partial pressure of oxygen
FiO2: Fraction of inspired oxygen
BMI: Body mass index
PBW: Predicted body weight
RR: Respiratory rate
CPAP: Continuous positive airway pressure
Pes: Esophageal pressure
Pl: Transpulmonary pressure
TIV: Tidal impedance variation
RS: Respiratory system
L: Lung
CW: Chest wall
Ppeak: Peak pressure
GI: Global inhomogeneity index
ΔPEEP: Difference between the set PEEP level and the EIT-based optimal PEEP
