# Supplementary material for: The “mechanical paradox” unveiled: a physiological study
Source: Crit Care. 2025 May 16;29:194. doi: 10.1186/s13054-025-05385-9 (PMC12082864; doi:10.1186/s13054-025-05385-9)
Supplement: Supplementary file 1 — Additional file 1. [file 13054_2025_5385_MOESM1_ESM.docx]

**Additional File 1. Ventilatory settings, intra-abdominal pressure, and respiratory mechanics**

| **Table E1: Ventilator settings on study entry** | | | | | | |
| --- | --- | --- | --- | --- | --- | --- |
| Patient | PaO_2_/FiO_2_ (mmHg) | Vt (mL) | RR (cycles/minute) | PEEP (cmH_2_O) | Cstat_RS (mL/cmH2O) | Optimal PEEP  (cmH_2_O) |
| 1 | 70 | 330 | 20 | 14 | 29 | 12 |
| 2 | 208 | 440 | 25 | 15 | 40 | 14 |
| 3 | 159 | 440 | 20 | 10 | 65 | 16 |
| 4 | 185 | 320 | 18 | 8 | 44 | 10 |
| 5 | 194 | 220 | 28 | 10 | 20 | 10 |
| 6 | 220 | 380 | 20 | 14 | 44 | 12 |
| 7 | 135 | 180 | 18 | 14 | 34 | 12 |
| 8 | 115 | 390 | 28 | 6 | 42 | 12 |
| 9 | 240 | 450 | 20 | 12 | 67 | 10 |
| 10 | 170 | 440 | 22 | 14 | 57 | 10 |
| 11 | 200 | 480 | 24 | 15 | 60 | 12 |
| 12 | 175 | 360 | 22 | 14 | 46 | 14 |
| 13 | 270 | 380 | 22 | 18 | 63 | 12 |
| 14 | 140 | 420 | 24 | 14 | 59 | 14 |
| 15 | 202 | 450 | 18 | 10 | 73 | 12 |
| 16 | 185 | 450 | 20 | 15 | 72 | 14 |
| 17 | 122 | 400 | 20 | 12 | 41 | 16 |
| 18 | 150 | 450 | 22 | 8 | 29 | 12 |
| 19 | 147 | 400 | 20 | 16 | 65 | 14 |
| 20 | 194 | 440 | 20 | 14 | 67 | 12 |
| *Abbreviations:* PaO_2_, arterial partial pressure of oxygen; FiO_2_, fraction of inspired oxygen; Vt, tidal volume; RR, respiratory rate; PEEP, positive end-expiratory pressure; Cstat_RS, static compliance of the respiratory system; optimal PEEP, value corresponding to the intersection of the curves representing the cumulative percentage of compliance loss due to either collapse or overdistension measured with electrical impedance tomography. | | | | | | |

| **Table E2: Variations in IAP at the various study phases and at different PEEP levels** | | | | | | | | | | | | | | | | | | | | | | |
| --- | --- | --- | --- | --- | --- | --- | --- | --- | --- | --- | --- | --- | --- | --- | --- | --- | --- | --- | --- | --- | --- | --- |
| Pt | PEEP | 20 | | | 18 | | | 16 | | | 14 | | | 12 | | | 10 | | | 8 | | |
|  | Phase | 1 | 2 | 3 | 1 | 2 | 3 | 1 | 2 | 3 | 1 | 2 | 3 | 1 | 2 | 3 | 1 | 2 | 3 | 1 | 2 | 3 |
| 1 | IAP | 7 | 12 | 7 | 7 | 12 | 7 | 8 | 13 | 7 | 7 | 12 | 7 | 7 | 12 | 7 | 7 | 12 | 7 | 7 | 12 | 6 |
|  | Weight | / | 5 | / | / | 5 | / | / | 7 | / | / | 6 | / | / | 7 | / | / | 7 | / | / | 8 | / |
| 2 | IAP | 14 | 19 | 15 | 15 | 20 | 14 | 14 | 19 | 14 | 14 | 19 | 14 | 14 | 19 | 14 | 14 | 19 | 13 | 13 | 18 | 13 |
|  | Weight | / | 5 | / | / | 8 | / | / | 7 | / | / | 7 | / | / | 7 | / | / | 7 | / | / | 6 | / |
| 3 | IAP | 13 | 18 | 13 | 13 | 18 | 13 | 13 | 18 | 13 | 12 | 17 | 12 | 12 | 17 | 12 | 12 | 17 | 12 | 12 | 17 | 12 |
|  | Weight | / | 8 | / | / | 8 | / | / | 8 | / | / | 8 | / | / | 8 | / | / | 9 | / | / | 8 | / |
| 4 | IAP | 15 | 20 | 14 | 13 | 18 | 13 | 13 | 18 | 13 | 13 | 18 | 13 | 13 | 18 | 13 | 13 | 18 | 13 | 12 | 17 | 12 |
|  | Weight | / | 6 | / | / | 6 | / | / | 6 | / | / | 7 | / | / | 7 | / | / | 7 | / | / | 6 | / |
| 5 | IAP | 7 | 12 | 7 | 7 | 12 | 7 | 7 | 12 | 7 | 7 | 12 | 7 | 7 | 12 | 7 | 7 | 12 | 7 | 7 | 12 | 7 |
|  | Weight | / | 8 | / | / | 8 | / | / | 9 | / | / | 8 | / | / | 8 | / | / | 7 | / | / | 8 | / |
| 6 | IAP | 10 | 15 | 9 | 9 | 14 | 9 | 9 | 14 | 9 | 9 | 14 | 9 | 8 | 13 | 8 | 8 | 13 | 8 | 8 | 13 | 8 |
|  | Weight | / | 8 | / | / | 7 | / | / | 8 | / | / | 8 | / | / | 9 | / | / | 9 | / | / | 9 | / |
| 7 | IAP | 12 | 17 | 11 | 10 | 15 | 10 | 10 | 15 | 10 | 10 | 15 | 10 | 10 | 15 | 10 | 9 | 14 | 9 | 9 | 14 | 9 |
|  | Weight | / | 7 | / | / | 7 | / | / | 7 | / | / | 7 | / | / | 7 | / | / | 7 | / | / | 7 | / |
| 8 | IAP | 16 | 21 | 16 | 16 | 21 | 16 | 16 | 21 | 16 | 15 | 20 | 16 | 16 | 21 | 17 | 17 | 22 | 16 | 16 | 21 | 16 |
|  | Weight | / | 7 | / | / | 7 | / | / | 7 | / | / | 7 | / | / | 8 | / | / | 8 | / | / | 7 | / |
| 9 | IAP | 15 | 20 | 16 | 15 | 20 | 15 | 15 | 20 | 15 | 14 | 19 | 14 | 14 | 19 | 14 | 14 | 19 | 14 | 14 | 19 | 13 |
|  | Weight | / | 8 | / | / | 9 | / | / | 9 | / | / | 9 | / | / | 10 | / | / | 10 | / | / | 12 | / |
| 10 | IAP | 15 | 20 | 15 | 15 | 20 | 15 | 15 | 20 | 15 | 14 | 19 | 14 | 14 | 19 | 14 | 14 | 19 | 13 | 13 | 18 | 13 |
|  | Weight | / | 9 | / | / | 9 | / | / | 9 | / | / | 9 | / | / | 9 | / | / | 10 | / | / | 9 | / |
| 11 | IAP | 12 | 17 | 10 | 11 | 16 | 10 | 10 | 15 | 11 | 10 | 15 | 10 | 10 | 15 | 10 | 10 | 15 | 10 | 10 | 15 | 10 |
|  | Weight | / | 9 | / | / | 8 | / | / | 7 | / | / | 6 | / | / | 7 | / | / | 8 | / | / | 8 | / |
| 12 | IAP | 16 | 21 | 17 | 16 | 21 | 16 | 16 | 21 | 18 | 18 | 23 | 18 | 18 | 23 | 18 | 18 | 23 | 18 | 18 | 23 | 18 |
|  | Weight | / | 7 | / | / | 7 | / | / | 7 | / | / | 7 | / | / | 7 | / | / | 6 | / | / | 6 | / |
| 13 | IAP | 11 | 16 | 10 | 10 | 15 | 10 | 9 | 14 | 10 | 8 | 13 | 9 | 10 | 15 | 10 | 10 | 15 | 10 | 11 | 16 | 11 |
|  | Weight | / | 7 | / | / | 8 | / | / | 7 | / | / | 9 | / | / | 9 | / | / | 8 | / | / | 9 | / |
| 14 | IAP | 12 | 17 | 13 | 13 | 18 | 13 | 13 | 18 | 13 | 13 | 18 | 13 | 13 | 18 | 13 | 13 | 18 | 13 | 12 | 17 | 12 |
|  | Weight | / | 9 | / | / | 12 | / | / | 12 | / | / | 13 | / | / | 12 | / | / | 12 | / | / | 10 | / |
| 15 | IAP | 9 | 14 | 9 | 9 | 14 | 9 | 9 | 14 | 9 | 9 | 14 | 9 | 8 | 13 | 9 | 8 | 13 | 8 | 8 | 13 | 9 |
|  | Weight | / | 8 | / | / | 8 | / | / | 8 | / | / | 8 | / | / | 7 | / | / | 8 | / | / | 8 | / |
| 16 | IAP | 12 | 17 | 14 | 13 | 18 | 13 | 12 | 17 | 12 | 12 | 17 | 12 | 12 | 17 | 11 | 11 | 16 | 11 | 10 | 15 | 10 |
|  | Weight | / | 6 | / | / | 6 | / | / | 9 | / | / | 11 | / | / | 12 | / | / | 12 | / | / | 11 | / |
| 17 | IAP | 19 | 24 | 19 | 18 | 23 | 18 | 19 | 24 | 19 | 19 | 24 | 19 | 18 | 23 | 18 | 18 | 23 | 18 | 18 | 23 | 18 |
|  | Weight | / | 7 | / | / | 7 | / | / | 7 | / | / | 7 | / | / | 7 | / | / | 6 | / | / | 7 | / |
| 18 | IAP | 14 | 19 | 13 | 13 | 18 | 13 | 13 | 18 | 13 | 13 | 18 | 13 | 13 | 18 | 13 | 12 | 17 | 12 | 12 | 17 | 12 |
|  | Weight | / | 7 | / | / | 6 | / | / | 7 | / | / | 7 | / | / | 7 | / | / | 7 | / | / | 6 | / |
| 19 | IAP | 8 | 13 | 8 | 8 | 13 | 8 | 7 | 12 | 7 | 7 | 12 | 7 | 7 | 12 | 7 | 6 | 11 | 6 | 6 | 11 | 6 |
|  | Weight | / | 7 | / | / | 7 | / | / | 7 | / | / | 7 | / | / | 7 | / | / | 7 | / | / | 7 | / |
| 20 | IAP | 7 | 12 | 7 | 7 | 12 | 6 | 6 | 11 | 5 | 5 | 10 | 5 | 5 | 10 | 5 | 5 | 10 | 5 | 5 | 10 | 5 |
|  | Weight | / | 8 | / | / | 9 | / | / | 9 | / | / | 9 | / | / | 9 | / | / | 8 | / | / | 8 | / |
| Phase 1, weight-off; phase 2, weight-on; phase 3, weight-off. PEEP is in cmH_2_O, IAP is in mmHg, weight in kg.  *Abbreviations:* Pt, patient; IAP, intra-abdominal pressure; PEEP, positive end-expiratory pressure. | | | | | | | | | | | | | | | | | | | | | | |

**Table E3: Variations of delta-PEEP 0 values for each patient across the three study phases**

| Patient | Phase 1 | Phase 2 | Phase 3 |
| --- | --- | --- | --- |
| 1 | 12 | 14 | 12 |
| 2 | 14 | 16 | 14 |
| 3 | 16 | 20 | 14 |
| 4 | 10 | 14 | 10 |
| 5 | 10 | 16 | 10 |
| 6 | 12 | 16 | 12 |
| 7 | 12 | 16 | 12 |
| 8 | 12 | 16 | 14 |
| 9 | 10 | 16 | 10 |
| 10 | 10 | 14 | 12 |
| 11 | 12 | 20 | 10 |
| 12 | 14 | 18 | 12 |
| 13 | 12 | 16 | 14 |
| 14 | 14 | 20 | 14 |
| 15 | 12 | 18 | 12 |
| 16 | 14 | 16 | 10 |
| 17 | 16 | 18 | 16 |
| 18 | 12 | 18 | 14 |
| 19 | 14 | 18 | 14 |
| 20 | 12 | 18 | 12 |
| Median (first quartile; third quartile) | 12 (12; 14) | 16 (16; 18) | 12 (11.5; 14) |
| Phase 1, weight-off; phase 2, weight-on; phase 3, weight-off. PEEP is reported in cmH_2_O. Delta-PEEP 0 is the EIT-based optimal PEEP measured at each study phase.  *Abbreviations:* PEEP, positive end-expiratory pressure. | | | |

**Figure E1: Variations of delta-PEEP 0 values across the three study phases**

Boxplots of the delta-PEEP 0 values across the three study phases, i.e., phase 1 (weight-off), phase 2 (weight-on), and phase 3 (weight-off), are presented. Each boxplot shows the median, 1^st^, and 3^rd^ quartile of delta-PEEP 0 values. Black dots represent each patient.

The Friedman test found a significant difference among the groups (p<0.001) with a large effect size (Kendall’s W=0.882). A post-hoc Wilcoxon signed-rank test with Bonferroni correction showed that delta-PEEP 0 at phase 2 was significantly higher than at both phase 1 (p=0.0002) and phase 3 (p=0.0002). No significant difference was observed between delta-PEEP 0 values at phase 1 and phase 3.

*Abbreviations:* PEEP, positive end-expiratory pressure.

| **Table E4: Mechanics of the respiratory system, lung, and chest wall for each study phase and ΔPEEP** | | | |
| --- | --- | --- | --- |
| ΔPEEP | Δ10^1^ | | |
| Phase | 1 | 2 | 3 |
| Ppeak_RS (cmH_2_O) | 48 [41-54] | 40 [33-50] | 49 [42-56] |
| Pplat_RS (cmH_2_O) | 35 [32-37] | 32 [29-36] | 38 [35-40] |
| Cstat_RS (mL x cmH_2_O^-1^) | 29 [21-38] | 43 [21-65] | 25 [16-36] |
| DP_RS (cmH_2_O) | 13 [12-15] | 10 [7-15] | 16 [13-19] |
| Pplat_L (cmH_2_O) | 17 [16-23] | 13 [9-19] | 20 [16-28] |
| Cstat_L (mL x cmH_2_O^-1^) | 46 [27-59] | 59 [27-100] | 39 [17-59] |
| DP_L (cmH_2_O) | 9 [8-12] | 8 [5-12] | 12 [8-18] |
| Pplat_CW (cmH_2_O) | 14 [12-16] | 18 [17-19] | 14 [12-16] |
| Cstat_CW (mL x cmH_2_O^-1^) | 232 [92-550] | 241 [110-375] | 252 [202-377] |
| ΔPEEP | Δ8^2^ | | |
| Phase | 1 | 2 | 3 |
| Ppeak_RS (cmH_2_O) | 41 [38-48] | 38 [35-40] | 41 [37-47] |
| Pplat_RS (cmH_2_O) | 30 [28-33] | 27 [26-30] | 30 [27-35] |
| Cstat_RS (mL x cmH_2_O^-1^) | 33 [26-58] | 48 [32-70] | 30 [27-56] |
| DP_RS (cmH_2_O) | 11 [7-13] | 6 [6-10] | 11 [7-14] |
| Pplat_L (cmH_2_O) | 16 [12-22] | 9 [6-14] | 15 [13-22] |
| Cstat_L (mL x cmH_2_O^-1^) | 42 [31-70] | 70 [42-96] | 39 [31-77] |
| DP_L (cmH_2_O) | 7 [6-11] | 5 [5-8] | 7 [6-11] |
| Pplat_CW (cmH_2_O) | 15 [11-19] | 17 [15-20] | 14 [12-16] |
| Cstat_CW (mL x cmH_2_O^-1^) | 321 [131-400] | 265 [132-346] | 244 [120-400] |
| ΔPEEP | Δ6^3^ | | |
| Phase | 1 | 2 | 3 |
| Ppeak_RS (cmH_2_O) | 37 [35-41] | 35 [34-37] | 37 [35-42] |
| Pplat_RS (cmH_2_O) | 29 [25-30] | 26 [24-28] | 27 [25-31] |
| Cstat_RS (mL x cmH_2_O^-1^) | 45 [30-61] | 59 [38-80] | 52 [31-66] |
| DP_RS (cmH_2_O) | 9 [6-12] | 6 [5-9] | 7 [6-12] |
| Pplat_L (cmH_2_O) | 12 [10-17] | 8 [6-11] | 13 [10-19] |
| Cstat_L (mL x cmH_2_O^-1^) | 67 [37-77] | 74 [55-114] | 72 [35-91] |
| DP_L (cmH_2_O) | 6 [5-9] | 5 [4-6] | 6 [5-9] |
| Pplat_CW (cmH_2_O) | 14 [12-18] | 19 [15-21] | 14 [12-17] |
| Cstat_CW (mL x cmH_2_O^-1^) | 230 [138-359] | 232 [138-350] | 230 [131-427] |
| ΔPEEP | Δ4 | | |
| Phase | 1 | 2 | 3 |
| Ppeak_RS (cmH_2_O) | 35 [33-38] | 34 [32-37] | 35 [32-38] |
| Pplat_RS (cmH_2_O) | 26 [23-28] | 25 [23-26] | 26 [23-28] |
| Cstat_RS (mL x cmH_2_O^-1^) | 46 [32-68] | 57 [40-81] | 51 [32-68] |
| DP_RS (cmH_2_O) | 8 [6-11] | 6 [5-9] | 8 [6-11] |
| Pplat_L (cmH_2_O) | 11 [8-15] | 6 [4-8] | 11 [9-16] |
| Cstat_L (mL x cmH_2_O^-1^) | 70 [38-94] | 86 [50-115] | 63 [39-89] |
| DP_L (cmH_2_O) | 6 [5-9] | 4 [4-8] | 6 [5-8] |
| Pplat_CW (cmH_2_O) | 14 [12-17] | 19 [16-21] | 14 [11-17] |
| Cstat_CW (mL x cmH_2_O^-1^) | 246 [157-409] | 291 [146-409] | 247 [176-536] |
| ΔPEEP | Δ2 | | |
| Phase | 1 | 2 | 3 |
| Ppeak_RS (cmH_2_O) | 32 [30-35] | 32 [31-36] | 32 [31-35] |
| Pplat_RS (cmH_2_O) | 23 [21-25] | 23 [21-24] | 24 [21-26] |
| Cstat_RS (mL x cmH_2_O^-1^) | 53 [34-80] | 60 [39-81] | 54 [34-75] |
| DP_RS (cmH_2_O) | 7 [5-10] | 6 [5-9] | 7 [5-11] |
| Pplat_L (cmH_2_O) | 10 [7-13] | 4 [2-8] | 10 [7-14] |
| Cstat_L (mL x cmH_2_O^-1^) | 66 [46-99] | 95 [51-112] | 73 [41-98] |
| DP_L (cmH_2_O) | 6 [4-8] | 4 [4-8] | 6 [4-8] |
| Pplat_CW (cmH_2_O) | 13 [10-16] | 18 [15-21] | 14 [10-16] |
| Cstat_CW (mL x cmH_2_O^-1^) | 266 [198-516] | 195 [139-338] | 244 [171-422] |
| ΔPEEP | Δ0 | | |
| Phase | 1 | 2 | 3 |
| Ppeak_RS (cmH_2_O) | 29 [28-33] | 31 [28-35] | 30 [29-34] |
| Pplat_RS (cmH_2_O) | 22 [19-23] | 21 [19-23] | 21 [19-23] |
| Cstat_RS (mL x cmH_2_O^-1^) | 50 [37-72] | 56 [38-73] | 57 [39-71] |
| DP_RS (cmH_2_O) | 7 [5-10] | 7 [6-9] | 7 [5-10] |
| Pplat_L (cmH_2_O) | 8 [6-12] | 3 [2-7] | 9 [6-11] |
| Cstat_L (mL x cmH_2_O^-1^) | 70 [45-107] | 83 [47-102] | 66 [48-92] |
| DP_L (cmH_2_O) | 5 [4-8] | 5 [4-8] | 6 [4-8] |
| Pplat_CW (cmH_2_O) | 13 [10-15] | 18 [15-20] | 12 [10-16] |
| Cstat_CW (mL x cmH_2_O^-1^) | 269 [174-347] | 210 [126-282] | 267 [183-432] |
| ΔPEEP | Δ-2 | | |
| Phase | 1 | 2 | 3 |
| Ppeak_RS (cmH_2_O) | 27 [26-31] | 30 [28-33] | 28 [27-32] |
| Pplat_RS (cmH_2_O) | 19 [17-21] | 19 [17-22] | 19 [17-21] |
| Cstat_RS (mL x cmH_2_O^-1^) | 57 [41-73] | 58 [40-68] | 54 [40-74] |
| DP_RS (cmH_2_O) | 7 [6-10] | 7 [6-10] | 7 [6-9] |
| Pplat_L (cmH_2_O) | 7 [5-9] | 3 [1-6] | 7 [5-10] |
| Cstat_L (mL x cmH_2_O^-1^) | 78 [47-93] | 77 [46-109] | 78 [44-93] |
| DP_L (cmH_2_O) | 5 [4-8] | 5 [4-8] | 5 [5-7] |
| Pplat_CW (cmH_2_O) | 12 [9-15] | 18 [13-19] | 12 [9-14] |
| Cstat_CW (mL x cmH_2_O^-1^) | 223 [155-492] | 247 [129-341] | 250 [143-380] |
| ΔPEEP | Δ-4^4^ | | |
| Phase | 1 | 2 | 3 |
| Ppeak_RS (cmH_2_O) | 27 [26-30] | 30 [29-35] | 27 [26-30] |
| Pplat_RS (cmH_2_O) | 18 [15-19] | 20 [17-22] | 18 [15-20] |
| Cstat_RS (mL x cmH_2_O^-1^) | 64 [40-72] | 48 [37-64] | 56 [40-74] |
| DP_RS (cmH_2_O) | 6 [5-9] | 8 [6-10] | 7 [5-10] |
| Pplat_L (cmH_2_O) | 6 [3-8] | 2 [1-6] | 7 [3-8] |
| Cstat_L (mL x cmH_2_O^-1^) | 81 [48-98] | 78 [40-83] | 77 [49-94] |
| DP_L (cmH_2_O) | 5 [4-8] | 6 [5-10] | 5 [4-8] |
| Pplat_CW (cmH_2_O) | 13 [10-14] | 18 [16-20] | 13 [10-15] |
| Cstat_CW (mL x cmH_2_O^-1^) | 300 [178-457] | 233 [126-367] | 293 [190-788] |
| ΔPEEP | Δ-6^5^ | | |
| Phase | 1 | 2 | 3 |
| Ppeak_RS (cmH_2_O) | 27 [24-30] | 31 [30-35] | 28 [24-30] |
| Pplat_RS (cmH_2_O) | 17 [16-19] | 21 [19-22] | 17 [16-20] |
| Cstat_RS (mL x cmH_2_O^-1^) | 65 [44-75] | 43 [38-66] | 56 [43-74] |
| DP_RS (cmH_2_O) | 7 [5-9] | 9 [6-11] | 8 [6-9] |
| Pplat_L (cmH_2_O) | 5 [4-7] | 2 [1-3] | 6 [4-7] |
| Cstat_L (mL x cmH_2_O^-1^) | 77 [54-100] | 60 [44-97] | 75 [49-93] |
| DP_L (cmH_2_O) | 6 [4-7] | 7 [4-9] | 6 [4-8] |
| Pplat_CW (cmH_2_O) | 14 [11-15] | 20 [18-20] | 13 [11-15] |
| Cstat_CW (mL x cmH_2_O^-1^) | 364 [227-425] | 267 [161-373] | 300 [233-582] |
| ΔPEEP | Δ-8^6^ | | |
| Phase | 1 | 2 | 3 |
| Ppeak_RS (cmH_2_O) | 27 [27-27] | 33 [32-33] | 27 [27-28] |
| Pplat_RS (cmH_2_O) | 18 [17-18] | 23 [22-24] | 18 [18-19] |
| Cstat_RS (mL x cmH_2_O^-1^) | 46 [43-50] | 30 [28-33] | 44 [41-47] |
| DP_RS (cmH_2_O) | 9 [9-10] | 14 [13-15] | 10 [9-10] |
| Pplat_L (cmH_2_O) | 3 [3-4] | 1 [1-1] | 4 [3-4] |
| Cstat_L (mL x cmH_2_O^-1^) | 59 [52-66] | 38 [33-43] | 59 [53-66] |
| DP_L (cmH_2_O) | 7 [7-8] | 12 [10-13] | 7 [7-8] |
| Pplat_CW (cmH_2_O) | 15 [15-15] | 22 [21-22] | 15 [15-15] |
| Cstat_CW (mL x cmH_2_O^-1^) | 233 [217-250] | 198 [164-232] | 180 [175-185] |
| Variables are expressed as median [1^st^-3^rd^ quartile].  Not all outcome variables could be measured for each ΔPEEP level because of the fixed PEEP range explored in the decremental PEEP trial and the varying best PEEP value for each patient. Here follow the patients with missing ΔPEEP values:  ^1^Missing data for 16 patients (ID 1,2,3,6,7,8,11,12,13,14,15,16,17,18,19,20)  ^2^Missing data for 7 patients (ID 2,3,12,14,16,17,19)  ^3^Missing data for 2 patients (ID 3,17)  ^4^Missing data for 4 patients (ID 4,5,9,10)  ^5^Missing data for 13 patients (ID 1,4,5,6,7,8,9,10,11,13,15,18,20)  ^6^Missing data for 18 patients (ID 1,2,4,5,6,7,8,9,10,11,12,13,14,15,16,18,19,20)  *Abbreviations:* PEEP, positive end-expiratory pressure; Ppeak, peak pressure; RS, respiratory system; Pplat, plateau pressure; Cstat, static compliance; DP, driving pressure; L, lung, CW; chest wall. | | | |

| **Table E5: Effect of different phases and PEEP levels on respiratory mechanics** | | | |
| --- | --- | --- | --- |
| Ppeak_RS (cmH_2_O) | | | |
| PEEP ≥ best PEEP | | | |
| Variable | Coefficient | Standard error | P-value |
| Phase 2 | -1.21 | 0.27 | <0.001 |
| Phase 3 | 0.21 | 0.27 | <0.001 |
| Δ2 | 1.74 | 0.34 | <0.001 |
| Δ4 | 3.91 | 0.34 | <0.001 |
| Δ6 | 6.46 | 0.35 | <0.001 |
| Δ8 | 10.10 | 0.39 | <0.001 |
| Δ10 | 15.16 | 0.62 | <0.001 |
| PEEP < best PEEP | | | |
| Variable | Coefficient | Standard error | P-value |
| Phase 2 | 3.33 | 0.29 | <0.001 |
| Phase 3 | 0.53 | 0.29 | 0.074 |
| Δ-2 | 2.36 | 0.69 | <0.001 |
| Δ-4 | 1.37 | 0.69 | 0.045 |
| Δ-6 | 0.35 | 0.70 | 0.620 |
| Pplat_RS (cmH_2_O) | | | |
| PEEP ≥ best PEEP | | | |
| Variable | Coefficient | Standard error | P-value |
| Phase 2 | -1.26 | 0.21 | <0.001 |
| Phase 3 | 0.09 | 0.21 | <0.001 |
| Δ2 | 1.95 | 0.27 | <0.001 |
| Δ4 | 4.26 | 0.27 | <0.001 |
| Δ6 | 6.79 | 0.28 | <0.001 |
| Δ8 | 9.98 | 0.31 | <0.001 |
| Δ10 | 14.80 | 0.50 | <0.001 |
| PEEP < best PEEP | | | |
| Variable | Coefficient | Standard error | P-value |
| Phase 2 | 2.09 | 0.20 | <0.001 |
| Phase 3 | 0.28 | 0.20 | 0.173 |
| Δ-2 | 3.21 | 0.47 | <0.001 |
| Δ-4 | 1.75 | 0.47 | <0.001 |
| Δ-6 | 0.52 | 0.49 | 0.290 |
| Cstat_RS (mL x cmH_2_O^-1^) | | | |
| PEEP ≥ best PEEP | | | |
| Variable | Coefficient | Standard error | P-value |
| Phase 2 | 7.36 | 1.10 | <0.001 |
| Phase 3 | 0.41 | 1.10 | 0.707 |
| Δ2 | 0.08 | 1.38 | 0.955 |
| Δ4 | -3.20 | 1.38 | 0.021 |
| Δ6 | -5.85 | 1.43 | <0.001 |
| Δ8 | -10.80 | 1.60 | <0.001 |
| Δ10 | -20.57 | 2.55 | <0.001 |
| PEEP < best PEEP | | | |
| Variable | Coefficient | Standard error | P-value |
| Phase 2 | -7.93 | 1.39 | <0.001 |
| Phase 3 | -1.59 | 1.39 | 0.256 |
| Δ-2 | 9.36 | 3.23 | 0.005 |
| Δ-4 | 8.42 | 3.23 | 0.010 |
| Δ-6 | 9.97 | 3.32 | 0.003 |
| DP_RS (cmH_2_O) | | | |
| PEEP ≥ best PEEP | | | |
| Variable | Coefficient | Standard error | P-value |
| Phase 2 | -1.47 | 0.22 | <0.001 |
| Phase 3 | 0.02 | 0.22 | 0.942 |
| Δ2 | 0.07 | 0.28 | 0.805 |
| Δ4 | 0.54 | 0.28 | 0.052 |
| Δ6 | 1.16 | 0.29 | <0.001 |
| Δ8 | 2.49 | 0.32 | <0.001 |
| Δ10 | 5.46 | 0.51 | <0.001 |
| PEEP < best PEEP | | | |
| Variable | Coefficient | Standard error | P-value |
| Phase 2 | 1.12 | 0.21 | <0.001 |
| Phase 3 | 0.20 | 0.21 | 0.345 |
| Δ-2 | -2.38 | 0.48 | <0.001 |
| Δ-4 | -2.12 | 0.48 | <0.001 |
| Δ-6 | -2.00 | 0.49 | <0.001 |
| Pplat_L (cmH_2_O) | | | |
| PEEP ≥ best PEEP | | | |
| Variable | Coefficient | Standard error | P-value |
| Phase 2 | -5.51 | 0.28 | <0.001 |
| Phase 3 | 0.34 | 0.28 | <0.001 |
| Δ2 | 1.41 | 0.35 | <0.001 |
| Δ4 | 3.15 | 0.35 | <0.001 |
| Δ6 | 5.12 | 0.36 | <0.001 |
| Δ8 | 7.62 | 0.40 | <0.001 |
| Δ10 | 11.32 | 0.64 | <0.001 |
| PEEP < best PEEP | | | |
| Variable | Coefficient | Standard error | P-value |
| Phase 2 | -3.35 | 0.28 | <0.001 |
| Phase 3 | 0.37 | 0.28 | 0.190 |
| Δ-2 | 2.96 | 0.65 | <0.001 |
| Δ-4 | 1.85 | 0.65 | 0.005 |
| Δ-6 | 1.26 | 0.67 | 0.006 |
| Cstat_L (mL x cmH_2_O^-1^) | | | |
| PEEP ≥ best PEEP | | | |
| Variable | Coefficient | Standard error | P-value |
| Phase 2 | 22.03 | 3.22 | <0.001 |
| Phase 3 | 1.45 | 3.22 | 0.652 |
| Δ2 | 4.90 | 4.05 | 0.227 |
| Δ4 | -3.65 | 4.05 | 0.368 |
| Δ6 | -6.08 | 4.19 | 0.148 |
| Δ8 | -12.26 | 4.68 | 0.009 |
| Δ10 | -38.56 | 7.47 | <0.001 |
| PEEP < best PEEP | | | |
| Variable | Coefficient | Standard error | P-value |
| Phase 2 | -6.35 | 3.45 | 0.069 |
| Phase 3 | -1.04 | 3.44 | 0.764 |
| Δ-2 | 10.72 | 7.92 | 0.179 |
| Δ-4 | 11.22 | 7.93 | 0.160 |
| Δ-6 | 8.65 | 8.15 | 0.291 |
| DP_L (cmH_2_O) | | | |
| PEEP ≥ best PEEP | | | |
| Variable | Coefficient | Standard error | P-value |
| Phase 2 | -1.62 | 0.22 | <0.001 |
| Phase 3 | 0.01 | 0.22 | 0.949 |
| Δ2 | -0.01 | 0.28 | 0.986 |
| Δ4 | 0.45 | 0.28 | 0.103 |
| Δ6 | 0.98 | 0.29 | <0.001 |
| Δ8 | 2.23 | 0.32 | <0.001 |
| Δ10 | 5.06 | 0.51 | <0.001 |
| PEEP < best PEEP | | | |
| Variable | Coefficient | Standard error | P-value |
| Phase 2 | 0.69 | 0.20 | 0.001 |
| Phase 3 | 0.20 | 0.20 | 0.335 |
| Δ-2 | -1.83 | 0.47 | <0.001 |
| Δ-4 | -1.64 | 0.47 | <0.001 |
| Δ-6 | -1.40 | 0.48 | 0.005 |
| Pplat_CW (cmH_2_O) | | | |
| PEEP ≥ best PEEP | | | |
| Variable | Coefficient | Standard error | P-value |
| Phase 2 | 4.25 | 0.20 | <0.001 |
| Phase 3 | -0.26 | 0.20 | 0.207 |
| Δ2 | 0.55 | 0.26 | 0.034 |
| Δ4 | 1.11 | 0.26 | <0.001 |
| Δ6 | 1.67 | 0.27 | <0.001 |
| Δ8 | 2.37 | 0.30 | <0.001 |
| Δ10 | 3.48 | 0.48 | <0.001 |
| PEEP < best PEEP | | | |
| Variable | Coefficient | Standard error | P-value |
| Phase 2 | 5.48 | 0.29 | <0.001 |
| Phase 3 | -0.08 | 0.28 | 0.767 |
| Δ-2 | 0.21 | 0.66 | 0.750 |
| Δ-4 | -0.11 | 0.66 | 0.865 |
| Δ-6 | -0.75 | 0.67 | 0.268 |
| Cstat_CW (mL x cmH_2_O^-1^) | | | |
| PEEP ≥ best PEEP | | | |
| Variable | Coefficient | Standard error | P-value |
| Phase 2 | -15.13 | 77.91 | 0.846 |
| Phase 3 | 77.19 | 77.91 | 0.323 |
| Δ2 | 69.35 | 98.04 | 0.480 |
| Δ4 | 21.23 | 98.04 | 0.829 |
| Δ6 | 36.40 | 101.14 | 0.719 |
| Δ8 | 0.94 | 112.08 | 0.993 |
| Δ10 | 2.57 | 176.61 | 0.988 |
| PEEP < best PEEP | | | |
| Variable | Coefficient | Standard error | P-value |
| Phase 2 | 78.42 | 158.24 | 0.621 |
| Phase 3 | 127.94 | 157.51 | 0.418 |
| Δ-2 | -62.12 | 354.13 | 0.861 |
| Δ-4 | 64.37 | 355.74 | 0.857 |
| Δ-6 | 302.52 | 368.28 | 0.413 |
| The coefficients, standard errors, and p-values of the association between ΔPEEP, i.e., the difference between the set PEEP level and the EIT-based optimal PEEP, and each outcome variable are obtained through the application of mixed-effects models, including the individual patient as a random factor and the weight and ΔPEEP as fixed factors.  Reference for covariate “phase” is phase 1. Reference for covariate “ΔPEEP” is ΔPEEP 0 cmH_2_O for values ≥ best PEEP and ΔPEEP -8 cmH_2_O for values < best PEEP.  *Abbreviations:* PEEP, positive end-expiratory pressure; Ppeak, peak pressure; RS, respiratory system, Pplat, plateau pressure; Cstat, static compliance; DP, driving pressure; L, lung; CW, chest wall. | | | |

| **Table E6: Pairwise comparison of respiratory mechanics variables between different ΔPEEP levels and study phases** | | | | | | | | | | | |
| --- | --- | --- | --- | --- | --- | --- | --- | --- | --- | --- | --- |
| Ppeak_RS (cmH_2_O) | | | | | | | | | | | |
| *Comparison within study phase: Phase 1* | | | | | | | | | | | |
| PEEP ≥ best PEEP | | | | | | | PEEP < best PEEP | | | | |
| Δ2 | | | | 0.003 | | | Δ-2 | | | <0.001 | |
| Δ4 | | | | <0.001 | | | Δ-4 | | | 0.014 | |
| Δ6 | | | | <0.001 | | | Δ-6 | | | 0.153 | |
| Δ8 | | | | <0.001 | | |  | | |  | |
| Δ10 | | | | <0.001 | | |  | | |  | |
| *Comparison within study phase: Phase 2* | | | | | | | | | | | |
| PEEP ≥ best PEEP | | | | | | | PEEP < best PEEP | | | | |
| Δ2 | | | | 0.001 | | | Δ-2 | | | 0.752 | |
| Δ4 | | | | <0.001 | | | Δ-4 | | | 0.973 | |
| Δ6 | | | | <0.001 | | | Δ-6 | | | 0.184 | |
| Δ8 | | | | <0.001 | | |  | | |  | |
| Δ10 | | | | <0.001 | | |  | | |  | |
| *Comparison within study phase: Phase 3* | | | | | | | | | | | |
| PEEP ≥ best PEEP | | | | | | | PEEP < best PEEP | | | | |
| Δ2 | | | | <0.001 | | | Δ-2 | | | <0.001 | |
| Δ4 | | | | <0.001 | | | Δ-4 | | | 0.012 | |
| Δ6 | | | | <0.001 | | | Δ-6 | | | 0.124 | |
| Δ8 | | | | <0.001 | | |  | | |  | |
| Δ10 | | | | <0.001 | | |  | | |  | |
| *Comparison within PEEP level* | | | | | | | | | | | |
| Δ-8 | | | | | Δ-6 | | | Δ-4 | | | |
| Phase | 1 | | | | Phase | | 1 | Phase | | | 1 |
| 2 | 0.009 | | | | 2 | | <0.001 | 2 | | | <0.001 |
| 3 | 0.501 | | | | 3 | | 0.415 | 3 | | | 0.465 |
| Δ-2 | | | | | Δ0 | | | Δ2 | | | |
| Phase | 1 | | | | Phase | | 1 | Phase | | | 1 |
| 2 | <0.001 | | | | 2 | | 0.079 | 2 | | | 0.419 |
| 3 | 0.129 | | | | 3 | | 0.701 | 3 | | | 0.042 |
| Δ4 | | | | | Δ6 | | | Δ8 | | | |
| Phase | 1 | | | | Phase | | 1 | Phase | | | 1 |
| 2 | 0.011 | | | | 2 | | <0.001 | 2 | | | <0.001 |
| 3 | 0.579 | | | | 3 | | 0.849 | 3 | | | 0.5 |
| Δ10 | | | | |  |  |  |  |  |  |  |
| Phase | 1 | | | |  |  |  |  |  |  |  |
| 2 | 0.038 | | | |  |  |  |  |  |  |  |
| 3 | 0.276 | | | |  |  |  |  |  |  |  |
| Pplat_RS (cmH_2_O) | | | | | | | | | | | |
| *Comparison within study phase: Phase 1* | | | | | | | | | | | |
| PEEP ≥ best PEEP | | | | | | PEEP < best PEEP | | | | | |
| Δ2 | | | | <0.001 | | Δ-2 | | | <0.001 | | |
| Δ4 | | | | <0.001 | | Δ-4 | | | <0.001 | | |
| Δ6 | | | | <0.001 | | Δ-6 | | | 0.034 | | |
| Δ8 | | | | <0.001 | |  | | |  | | |
| Δ10 | | | | <0.001 | |  | | |  | | |
| *Comparison within study phase: Phase 2* | | | | | | | | | | | |
| PEEP ≥ best PEEP | | | | | | PEEP < best PEEP | | | | | |
| Δ2 | | | | <0.001 | | Δ-2 | | | 0.198 | | |
| Δ4 | | | | <0.001 | | Δ-4 | | | 0.901 | | |
| Δ6 | | | | <0.001 | | Δ-6 | | | 0.037 | | |
| Δ8 | | | | <0.001 | |  | | |  | | |
| Δ10 | | | | <0.001 | |  | | |  | | |
| *Comparison within study phase: Phase 3* | | | | | | | | | | | |
| PEEP ≥ best PEEP | | | | | | PEEP < best PEEP | | | | | |
| Δ2 | | | | <0.001 | | Δ-2 | | | <0.001 | | |
| Δ4 | | | | <0.001 | | Δ-4 | | | <0.001 | | |
| Δ6 | | | | <0.001 | | Δ-6 | | | 0.024 | | |
| Δ8 | | | | <0.001 | |  | | |  | | |
| Δ10 | | | | <0.001 | |  | | |  | | |
| *Comparison within PEEP level* | | | | | | | | | | | |
| Δ-8 | | | | | Δ-6 | | | Δ-4 | | | |
| Phase | 1 | | | | Phase | | 1 | Phase | | | 1 |
| 2 | 0.003 | | | | 2 | | <0.001 | 2 | | | <0.001 |
| 3 | 0.176 | | | | 3 | | 0.717 | 3 | | | 0.445 |
| Δ-2 | | | | | Δ0 | | | Δ2 | | | |
| Phase | 1 | | | | Phase | | 1 | Phase | | | 1 |
| 2 | <0.001 | | | | 2 | | 0.862 | 2 | | | 0.344 |
| 3 | 0.223 | | | | 3 | | 0.347 | 3 | | | 0.113 |
| Δ4 | | | | | Δ6 | | | Δ8 | | | |
| Phase | | | 1 | | Phase | | 1 | Phase | | | 1 |
| 2 | | | <0.001 | | 2 | | <0.001 | 2 | | | <0.001 |
| 3 | | | 0.960 | | 3 | | 0.913 | 3 | | | 0.765 |
| Δ10 | | | | |  |  |  |  |  |  |  |
| Phase | | | 1 | |  |  |  |  |  |  |  |
| 2 | | | 0.063 | |  |  |  |  |  |  |  |
| 3 | | | 0.920 | |  |  |  |  |  |  |  |
| Cstat_RS (mL x cmH_2_O^-1^) | | | | | | | | | | | |
| *Comparison within study phase: Phase 1* | | | | | | | | | | | |
| PEEP ≥ best PEEP | | | | | | PEEP < best PEEP | | | | | |
| Δ2 | | | | 0.970 | | Δ-2 | | | 0.383 | | |
| Δ4 | | | | 0.006 | | Δ-4 | | | 0.406 | | |
| Δ6 | | | | <0.001 | | Δ-6 | | | 0.109 | | |
| Δ8 | | | | <0.001 | |  | | |  | | |
| Δ10 | | | | <0.001 | |  | | |  | | |
| *Comparison within study phase: Phase 2* | | | | | | | | | | | |
| PEEP ≥ best PEEP | | | | | | PEEP < best PEEP | | | | | |
| Δ2 | | | | 0.255 | | Δ-2 | | | <0.001 | | |
| Δ4 | | | | 0.046 | | Δ-4 | | | 0.001 | | |
| Δ6 | | | | 0.086 | | Δ-6 | | | 0.001 | | |
| Δ8 | | | | 0.527 | |  | | |  | | |
| Δ10 | | | | <0.001 | |  | | |  | | |
| *Comparison within study phase: Phase 3* | | | | | | | | | | | |
| PEEP ≥ best PEEP | | | | | | PEEP < best PEEP | | | | | |
| Δ2 | | | | 0.404 | | Δ-2 | | | 0.192 | | |
| Δ4 | | | | 0.002 | | Δ-4 | | | 0.126 | | |
| Δ6 | | | | <0.001 | | Δ-6 | | | 0.216 | | |
| Δ8 | | | | <0.001 | |  | | |  | | |
| Δ10 | | | | <0.001 | |  | | |  | | |
| *Comparison within PEEP level* | | | | | | | | | | | |
| Δ-8 | | | | | Δ-6 | | | Δ-4 | | | |
| Phase | 1 | | | | Phase | | 1 | Phase | | | 1 |
| 2 | 0.014 | | | | 2 | | 0.014 | 2 | | | <0.001 |
| 3 | 0.356 | | | | 3 | | 0.371 | 3 | | | 0.908 |
| Δ-2 | | | | | Δ0 | | | Δ2 | | | |
| Phase | 1 | | | | Phase | | 1 | Phase | | | 1 |
| 2 | 0.049 | | | | 2 | | 0.852 | 2 | | | 0.435 |
| 3 | 0.486 | | | | 3 | | 0.813 | 3 | | | 0.471 |
| Δ4 | | | | | Δ6 | | | Δ8 | | | |
| Phase | 1 | | | | Phase | | 1 | Phase | | | 1 |
| 2 | <0.001 | | | | 2 | | <0.001 | 2 | | | <0.001 |
| 3 | 0.966 | | | | 3 | | 0.318 | 3 | | | 0.708 |
| Δ10 | | | | |  |  |  |  |  |  |  |
| Phase | | 1 | | |  |  |  |  |  |  |  |
| 2 | | 0.076 | | |  |  |  |  |  |  |  |
| 3 | | 0.703 | | |  |  |  |  |  |  |  |
| DP_RS (cmH_2_O) | | | | | | | | | | | |
| *Comparison within study phase: Phase 1* | | | | | | | | | | | |
| PEEP ≥ best PEEP | | | | | | PEEP < best PEEP | | | | | |
| Δ2 | | | | 0.971 | | Δ-2 | | | 0.041 | | |
| Δ4 | | | | 0.035 | | Δ-4 | | | 0.051 | | |
| Δ6 | | | | <0.001 | | Δ-6 | | | 0.057 | | |
| Δ8 | | | | <0.001 | |  | | |  | | |
| Δ10 | | | | <0.001 | |  | | |  | | |
| *Comparison within study phase: Phase 2* | | | | | | | | | | | |
| PEEP ≥ best PEEP | | | | | | PEEP < best PEEP | | | | | |
| Δ2 | | | | 0.331 | | Δ-2 | | | <0.001 | | |
| Δ4 | | | | 0.187 | | Δ-4 | | | <0.001 | | |
| Δ6 | | | | 0.408 | | Δ-6 | | | <0.001 | | |
| Δ8 | | | | 0.068 | |  | | |  | | |
| Δ10 | | | | <0.001 | |  | | |  | | |
| *Comparison within study phase: Phase 3* | | | | | | | | | | | |
| PEEP ≥ best PEEP | | | | | | PEEP < best PEEP | | | | | |
| Δ2 | | | | 0.322 | | Δ-2 | | | 0.097 | | |
| Δ4 | | | | 0.029 | | Δ-4 | | | 0.127 | | |
| Δ6 | | | | <0.001 | | Δ-6 | | | 0.535 | | |
| Δ8 | | | | <0.001 | |  | | |  | | |
| Δ10 | | | | <0.001 | |  | | |  | | |
| *Comparison within PEEP level* | | | | | | | | | | | |
| Δ-8 | | | | | Δ-6 | | | Δ-4 | | | |
| Phase | | 1 | | | Phase | | 1 | Phase | | | 1 |
| 2 | | 0.008 | | | 2 | | 0.01 | 2 | | | <0.001 |
| 3 | | 0.405 | | | 3 | | 0.189 | 3 | | | 0.689 |
| Δ-2 | | | | | Δ0 | | | Δ2 | | | |
| Phase | | 1 | | | Phase | | 1 | Phase | | | 1 |
| 2 | | 0.108 | | | 2 | | 0.493 | 2 | | | 0.091 |
| 3 | | 0.806 | | | 3 | | 0.459 | 3 | | | 0.429 |
| Δ4 | | | | | Δ6 | | | Δ8 | | | |
| Phase | | 1 | | | Phase | | 1 | Phase | | | 1 |
| 2 | | <0.001 | | | 2 | | <0.001 | 2 | | | <0.001 |
| 3 | | 0.794 | | | 3 | | 0.792 | 3 | | | 0.773 |
| Δ10 | | | | |  |  |  |  |  |  |  |
| Phase | | 1 | | |  |  |  |  |  |  |  |
| 2 | | 0.076 | | |  |  |  |  |  |  |  |
| 3 | | 0.108 | | |  |  |  |  |  |  |  |
| Pplat_L (cmH_2_O) | | | | | | | | | | | |
| *Comparison within study phase: Phase 1* | | | | | | | | | | | |
| PEEP ≥ best PEEP | | | | | | PEEP < best PEEP | | | | | |
| Δ2 | | | | 0.012 | | Δ-2 | | | <0.001 | | |
| Δ4 | | | | <0.001 | | Δ-4 | | | <0.001 | | |
| Δ6 | | | | <0.001 | | Δ-6 | | | 0.023 | | |
| Δ8 | | | | <0.001 | |  | | |  | | |
| Δ10 | | | | <0.001 | |  | | |  | | |
| *Comparison within study phase: Phase 2* | | | | | | | | | | | |
| PEEP ≥ best PEEP | | | | | | PEEP < best PEEP | | | | | |
| Δ2 | | | | 0.016 | | Δ-2 | | | 0.028 | | |
| Δ4 | | | | <0.001 | | Δ-4 | | | 0.252 | | |
| Δ6 | | | | <0.001 | | Δ-6 | | | 0.318 | | |
| Δ8 | | | | <0.001 | |  | | |  | | |
| Δ10 | | | | <0.001 | |  | | |  | | |
| *Comparison within study phase: Phase 3* | | | | | | | | | | | |
| PEEP ≥ best PEEP | | | | | | PEEP < best PEEP | | | | | |
| Δ2 | | | | 0.001 | | Δ-2 | | | <0.001 | | |
| Δ4 | | | | <0.001 | | Δ-4 | | | <0.001 | | |
| Δ6 | | | | <0.001 | | Δ-6 | | | 0.022 | | |
| Δ8 | | | | <0.001 | |  | | |  | | |
| Δ10 | | | | <0.001 | |  | | |  | | |
| *Comparison within PEEP level* | | | | | | | | | | | |
| Δ-8 | | | | | Δ-6 | | | Δ-4 | | | |
| Phase | | 1 | | | Phase | | 1 | Phase | | | 1 |
| 2 | | 0.115 | | | 2 | | <0.001 | 2 | | | <0.001 |
| 3 | | 0.749 | | | 3 | | 0.515 | 3 | | | 0.664 |
| Δ-2 | | | | | Δ0 | | | Δ2 | | | |
| Phase | | | 1 | | Phase | | 1 | Phase | | | 1 |
| 2 | | | <0.001 | | 2 | | <0.001 | 2 | | | <0.001 |
| 3 | | | 0.327 | | 3 | | 0.976 | 3 | | | 0.444 |
| Δ4 | | | | | Δ6 | | | Δ8 | | | |
| Phase | | | 1 | | Phase | | 1 | Phase | | | 1 |
| 2 | | | <0.001 | | 2 | | <0.001 | 2 | | | <0.001 |
| 3 | | | 0.468 | | 3 | | 0.757 | 3 | | | 0.704 |
| Δ10 | | | | |  |  |  |  |  |  |  |
| Phase | | 1 | | |  |  |  |  |  |  |  |
| 2 | | <0.001 | | |  |  |  |  |  |  |  |
| 3 | | 0.081 | | |  |  |  |  |  |  |  |
| Cstat_L (mL x cmH_2_O^-1^) | | | | | | | | | | | |
| *Comparison within study phase: Phase 1* | | | | | | | | | | | |
| PEEP ≥ best PEEP | | | | | | PEEP < best PEEP | | | | | |
| Δ2 | | | | 0.505 | | Δ-2 | | | 0.367 | | |
| Δ4 | | | | 0.158 | | Δ-4 | | | 0.496 | | |
| Δ6 | | | | 0.021 | | Δ-6 | | | 0.321 | | |
| Δ8 | | | | <0.001 | |  | | |  | | |
| Δ10 | | | | <0.001 | |  | | |  | | |
| *Comparison within study phase: Phase 2* | | | | | | | | | | | |
| PEEP ≥ best PEEP | | | | | | PEEP < best PEEP | | | | | |
| Δ2 | | | | 0.053 | | Δ-2 | | | 0.023 | | |
| Δ4 | | | | 0.336 | | Δ-4 | | | 0.073 | | |
| Δ6 | | | | 0.480 | | Δ-6 | | | 0.079 | | |
| Δ8 | | | | 0.482 | |  | | |  | | |
| Δ10 | | | | 0.009 | |  | | |  | | |
| *Comparison within study phase: Phase 3* | | | | | | | | | | | |
| PEEP ≥ best PEEP | | | | | | PEEP < best PEEP | | | | | |
| Δ2 | | | | 0.539 | | Δ-2 | | | 0.898 | | |
| Δ4 | | | | 0.037 | | Δ-4 | | | 0.533 | | |
| Δ6 | | | | 0.039 | | Δ-6 | | | 0.986 | | |
| Δ8 | | | | <0.001 | |  | | |  | | |
| Δ10 | | | | <0.001 | |  | | |  | | |
| *Comparison within PEEP level* | | | | | | | | | | | |
| Δ-8 | | | | | Δ-6 | | | Δ-4 | | | |
| Phase | | | 1 | | Phase | | 1 | Phase | | | 1 |
| 2 | | | 0.018 | | 2 | | 0.021 | 2 | | | 0.386 |
| 3 | | | 0.983 | | 3 | | 0.073 | 3 | | | 0.242 |
| Δ-2 | | | | | Δ0 | | | Δ2 | | | |
| Phase | | | 1 | | Phase | | 1 | Phase | | | 1 |
| 2 | | | 0.549 | | 2 | | 0.072 | 2 | | | 0.019 |
| 3 | | | 0.343 | | 3 | | 0.484 | 3 | | | 0.684 |
| Δ4 | | | | | Δ6 | | | Δ8 | | | |
| Phase | | | 1 | | Phase | | 1 | Phase | | | 1 |
| 2 | | | <0.001 | | 2 | | <0.001 | 2 | | | 0.012 |
| 3 | | | 0.954 | | 3 | | 0.389 | 3 | | | 0.822 |
| Δ10 | | | | |  |  |  |  |  |  |  |
| Phase | | | 1 | |  |  |  |  |  |  |  |
| 2 | | | 0.126 | |  |  |  |  |  |  |  |
| 3 | | | 0.851 | |  |  |  |  |  |  |  |
| DP_L (cmH_2_O) | | | | | | | | | | | |
| *Comparison within study phase: Phase 1* | | | | | | | | | | | |
| PEEP ≥ best PEEP | | | | | | PEEP < best PEEP | | | | | |
| Δ2 | | | | 0.728 | | Δ-2 | | | 0.142 | | |
| Δ4 | | | | 0.056 | | Δ-4 | | | 0.185 | | |
| Δ6 | | | | <0.001 | | Δ-6 | | | 0.257 | | |
| Δ8 | | | | <0.001 | |  | | |  | | |
| Δ10 | | | | <0.001 | |  | | |  | | |
| *Comparison within study phase: Phase 2* | | | | | | | | | | | |
| PEEP ≥ best PEEP | | | | | | PEEP < best PEEP | | | | | |
| Δ2 | | | | 0.16 | | Δ-2 | | | <0.001 | | |
| Δ4 | | | | 0.281 | | Δ-4 | | | <0.001 | | |
| Δ6 | | | | 0.641 | | Δ-6 | | | <0.001 | | |
| Δ8 | | | | 0.166 | |  | | |  | | |
| Δ10 | | | | <0.001 | |  | | |  | | |
| *Comparison within study phase: Phase 3* | | | | | | | | | | | |
| PEEP ≥ best PEEP | | | | | | PEEP < best PEEP | | | | | |
| Δ2 | | | | 0.503 | | Δ-2 | | | 0.566 | | |
| Δ4 | | | | 0.069 | | Δ-4 | | | 0.519 | | |
| Δ6 | | | | 0.003 | | Δ-6 | | | 0.809 | | |
| Δ8 | | | | <0.001 | |  | | |  | | |
| Δ10 | | | | <0.001 | |  | | |  | | |
| *Comparison within PEEP level* | | | | | | | | | | | |
| Δ-8 | | | | | Δ-6 | | | Δ-4 | | | |
| Phase | | | 1 | | Phase | | 1 | Phase | | | 1 |
| 2 | | | 0.049 | | 2 | | 0.084 | 2 | | | 0.004 |
| 3 | | | 0.963 | | 3 | | 0.179 | 3 | | | 0.968 |
| Δ-2 | | | | | Δ0 | | | Δ2 | | | |
| Phase | | | 1 | | Phase | | 1 | Phase | | | 1 |
| 2 | | | 0.537 | | 2 | | 0.121 | 2 | | | <0.001 |
| 3 | | | 0.486 | | 3 | | 0.701 | 3 | | | 0.829 |
| Δ4 | | | | | Δ6 | | | Δ8 | | | |
| Phase | | | 1 | | Phase | | 1 | Phase | | | 1 |
| 2 | | | <0.001 | | 2 | | <0.001 | 2 | | | <0.001 |
| 3 | | | 0.873 | | 3 | | 0.831 | 3 | | | 0.536 |
| Δ10 | | | | |  |  |  |  |  |  |  |
| Phase | | | 1 | |  |  |  |  |  |  |  |
| 2 | | | 0.129 | |  |  |  |  |  |  |  |
| 3 | | | 0.092 | |  |  |  |  |  |  |  |
| Pplat_CW (cmH_2_O) | | | | | | | | | | | |
| *Comparison within study phase: Phase 1* | | | | | | | | | | | |
| PEEP ≥ best PEEP | | | | | | PEEP < best PEEP | | | | | |
| Δ2 | | | | 0.216 | | Δ-2 | | | 0.039 | | |
| Δ4 | | | | <0.001 | | Δ-4 | | | 0.491 | | |
| Δ6 | | | | <0.001 | | Δ-6 | | | 0.772 | | |
| Δ8 | | | | <0.001 | |  | | |  | | |
| Δ10 | | | | <0.001 | |  | | |  | | |
| *Comparison within study phase: Phase 2* | | | | | | | | | | | |
| PEEP ≥ best PEEP | | | | | | PEEP < best PEEP | | | | | |
| Δ2 | | | | 0.096 | | Δ-2 | | | 0.524 | | |
| Δ4 | | | | 0.008 | | Δ-4 | | | 0.358 | | |
| Δ6 | | | | <0.001 | | Δ-6 | | | 0.015 | | |
| Δ8 | | | | <0.001 | |  | | |  | | |
| Δ10 | | | | <0.001 | |  | | |  | | |
| *Comparison within study phase: Phase 3* | | | | | | | | | | | |
| PEEP ≥ best PEEP | | | | | | PEEP < best PEEP | | | | | |
| Δ2 | | | | 0.023 | | Δ-2 | | | 0.439 | | |
| Δ4 | | | | <0.001 | | Δ-4 | | | 0.959 | | |
| Δ6 | | | | <0.001 | | Δ-6 | | | 0.462 | | |
| Δ8 | | | | <0.001 | |  | | |  | | |
| Δ10 | | | | <0.001 | |  | | |  | | |
| *Comparison within PEEP level* | | | | | | | | | | | |
| Δ-8 | | | | | Δ-6 | | | Δ-4 | | | |
| Phase | | | 1 | | Phase | | 1 | Phase | | | 1 |
| 2 | | | 0.004 | | 2 | | <0.001 | 2 | | | <0.001 |
| 3 | | | 0.802 | | 3 | | 0.678 | 3 | | | 0.985 |
| Δ-2 | | | | | Δ0 | | | Δ2 | | | |
| Phase | | | 1 | | Phase | | 1 | Phase | | | 1 |
| 2 | | | <0.001 | | 2 | | <0.001 | 2 | | | <0.001 |
| 3 | | | 0.739 | | 3 | | 0.496 | 3 | | | 0.962 |
| Δ4 | | | | | Δ6 | | | Δ8 | | | |
| Phase | | | 1 | | Phase | | 1 | Phase | | | 1 |
| 2 | | | <0.001 | | 2 | | <0.001 | 2 | | | <0.001 |
| 3 | | | 0.384 | | 3 | | 0.575 | 3 | | | 0.36 |
| Δ10 | | | | |  |  |  |  |  |  |  |
| Phase | | | 1 | |  |  |  |  |  |  |  |
| 2 | | | <0.001 | |  |  |  |  |  |  |  |
| 3 | | | 0.831 | |  |  |  |  |  |  |  |
| Cstat_CW (mL x cmH_2_O^-1^) | | | | | | | | | | | |
| *Comparison within study phase: Phase 1* | | | | | | | | | | | |
| PEEP ≥ best PEEP | | | | | | PEEP < best PEEP | | | | | |
| Δ2 | | | | 0.751 | | Δ-2 | | | 0.991 | | |
| Δ4 | | | | 0.726 | | Δ-4 | | | 0.970 | | |
| Δ6 | | | | 0.820 | | Δ-6 | | | 0.368 | | |
| Δ8 | | | | 0.962 | |  | | |  | | |
| Δ10 | | | | 0.819 | |  | | |  | | |
| *Comparison within study phase: Phase 2* | | | | | | | | | | | |
| PEEP ≥ best PEEP | | | | | | PEEP < best PEEP | | | | | |
| Δ2 | | | | 0.927 | | Δ-2 | | | 0.811 | | |
| Δ4 | | | | 0.729 | | Δ-4 | | | 0.887 | | |
| Δ6 | | | | 0.238 | | Δ-6 | | | 0.758 | | |
| Δ8 | | | | 0.392 | |  | | |  | | |
| Δ10 | | | | 0.682 | |  | | |  | | |
| *Comparison within study phase: Phase 3* | | | | | | | | | | | |
| PEEP ≥ best PEEP | | | | | | PEEP < best PEEP | | | | | |
| Δ2 | | | | 0.393 | | Δ-2 | | | 0.918 | | |
| Δ4 | | | | 0.433 | | Δ-4 | | | 0.603 | | |
| Δ6 | | | | 0.672 | | Δ-6 | | | 0.353 | | |
| Δ8 | | | | 0.409 | |  | | |  | | |
| Δ10 | | | | 0.631 | |  | | |  | | |
| *Comparison within PEEP level* | | | | | | | | | | | |
| Δ-8 | | | | | Δ-6 | | | Δ-4 | | | |
| Phase | | | 1 | | Phase | | 1 | Phase | | | 1 |
| 2 | | | 0.483 | | 2 | | 0.986 | 2 | | | 0.876 |
| 3 | | | 0.325 | | 3 | | 0.342 | 3 | | | 0.307 |
| Δ-2 | | | | | Δ0 | | | Δ2 | | | |
| Phase | | | 1 | | Phase | | 1 | Phase | | | 1 |
| 2 | | | 0.519 | | 2 | | 0.845 | 2 | | | 0.453 |
| 3 | | | 0.555 | | 3 | | 0.803 | 3 | | | 0.469 |
| Δ4 | | | | | Δ6 | | | Δ8 | | | |
| Phase | | | 1 | | Phase | | 1 | Phase | | | 1 |
| 2 | | | 0.663 | | 2 | | 0.925 | 2 | | | 0.487 |
| 3 | | | 0.088 | | 3 | | 0.671 | 3 | | | 0.819 |
| Δ10 | | | | |  |  |  |  |  |  |  |
| Phase | | | 1 | |  |  |  |  |  |  |  |
| 2 | | | 0.286 | |  |  |  |  |  |  |  |
| 3 | | | 0.579 | |  |  |  |  |  |  |  |
| Linear mixed-effects models were conducted for each ΔPEEP level. i.e., the difference between the set PEEP level and the EIT-based optimal PEEP, and study phase for pairwise comparisons relative to the effect of weight application and PEEP, respectively. In the comparison within phase reference for PEEP ≥ best PEEP is ΔPEEP=0; reference for PEEP > best PEEP is ΔPEEP = -8.  *Abbreviations:* PEEP, positive end-expiratory pressure; Ppeak, peak pressure; RS, respiratory system; Pplat, plateau pressure; Cstat, static compliance; DP, driving pressure; L, lung; CW, chest wall. | | | | | | | | | | | |
