# Supplementary material for: The “mechanical paradox” unveiled: a physiological study
Source: Crit Care. 2025 May 16;29:194. doi: 10.1186/s13054-025-05385-9 (PMC12082864; doi:10.1186/s13054-025-05385-9)
Supplement: Supplementary file 2 — Additional file 2. [file 13054_2025_5385_MOESM2_ESM.docx]

**Additional File 2. Electrical impedance tomography variables**

| **Table E7: EIT variables for each study phase and ΔPEEP** | | | |
| --- | --- | --- | --- |
| ΔPEEP | Δ10^1^ | | |
| Phase | 1 | 2 | 3 |
| OD_tot (%) | 25 [16-33] | 30 [22-35] | 25 [18-30] |
| CL_tot (%) | 0 [0-0.25] | 0 [0-1] | 0 [0-1] |
| GI_tot (%) | 51 [51-52] | 53 [50-55] | 51 [49-53] |
| ΔEELI_tot (%) | 557 [476-612] | 427 [392-447] | 568 [481-617] |
| ΔPEEP | Δ8^2^ | | |
| Phase | 1 | 2 | 3 |
| OD_tot (%) | 24 [19-29] | 18 [12-21] | 24 [19-28] |
| CL_tot (%) | 0 [0-1] | 8 [5-10] | 1 [0-1] |
| GI_tot (%) | 52 [49-54] | 51 [48-59] | 52 [48-55] |
| ΔEELI_tot (%) | 566 [487-731] | 393 [349-509] | 581 [466-733] |
| ΔPEEP | Δ6^3^ | | |
| Phase | 1 | 2 | 3 |
| OD_tot (%) | 23 [19-28] | 15 [10-19] | 22 [18-26] |
| CL_tot (%) | 1 [0-2] | 11 [6-12] | 2 [0-3] |
| GI_tot (%) | 50 [46-54] | 54 [47-59] | 51 [47-54] |
| ΔEELI_tot (%) | 551 [443-686] | 344 [286-438] | 533 [409-676] |
| ΔPEEP | Δ4 | | |
| Phase | 1 | 2 | 3 |
| OD_tot (%) | 20 [16-24] | 12 [9-14] | 18 [13-23] |
| CL_tot (%) | 4 [2-5] | 16 [13-19] | 5 [3-6] |
| GI_tot (%) | 51 [45-57] | 57 [50-67] | 51 [46-56] |
| ΔEELI_tot (%) | 461 [349-584] | 241 [185-300] | 451 [312-587] |
| ΔPEEP | Δ2 | | |
| Phase | 1 | 2 | 3 |
| OD_tot (%) | 16 [13-19] | 8 [6-10] | 14 [12-17] |
| CL_tot (%) | 6 [4-9] | 20 [16-24] | 8 [6-10] |
| GI_tot (%) | 51 [46-58] | 63 [53-68] | 52 [48-56] |
| ΔEELI_tot (%) | 397 [250-515] | 164 [111-204] | 359 [230-403] |
| ΔPEEP | Δ0 | | |
| Phase | 1 | 2 | 3 |
| OD_tot (%) | 11 [10-15] | 5 [2-6] | 10 [7-14] |
| CL_tot (%) | 11 [8-13] | 23 [22-27] | 13 [9-15] |
| GI_tot (%) | 52 [47-59] | 66 [59-73] | 54 [49-60] |
| ΔEELI_tot (%) | 316 [181-353] | 92 [55-119] | 276 [155-320] |
| ΔPEEP | Δ-2 | | |
| Phase | 1 | 2 | 3 |
| OD_tot (%) | 7 [5-9] | 1 [1-3] | 7 [4-8] |
| CL_tot (%) | 14 [12-18] | 27 [24-32] | 16 [12-18] |
| GI_tot (%) | 56 [50-61] | 69 [62-78] | 56 [52-64] |
| ΔEELI_tot (%) | 222 [111-270] | 40 [17-64] | 180 [92-236] |
| ΔPEEP | Δ-4^4^ | | |
| Phase | 1 | 2 | 3 |
| OD_tot (%) | 4 [2-7] | 1 [0-3] | 3 [1-5] |
| CL_tot (%) | 17 [15-19] | 30 [27-33] | 19 [16-24] |
| GI_tot (%) | 61 [52-69] | 76 [65-86] | 61 [53-71] |
| ΔEELI_tot (%) | 134 [89-200] | 0 [0-37] | 109 [61-159] |
| ΔPEEP | Δ-6^5^ | | |
| Phase | 1 | 2 | 3 |
| OD_tot (%) | 3 [2-8] | 0 [0-2] | 2 [2-6] |
| CL_tot (%) | 20 [19-21] | 32 [26-34] | 21 [21-22] |
| GI_tot (%) | 60 [57-67] | 71 [70-82] | 62 [56-68] |
| ΔEELI_tot (%) | 86 [37-151] | 0 [0-10] | 65 [19-119] |
| ΔPEEP | Δ-8^6^ | | |
| Phase | 1 | 2 | 3 |
| OD_tot (%) | 5 [4-5] | 4 [2-6] | 2 [1-2] |
| CL_tot (%) | 24 [24-25] | 29 [28-31] | 25 [24-25] |
| GI_tot (%) | 104 [85-122] | 124 [101-146] | 110 [89-130] |
| ΔEELI_tot (%) | -35 [-68-1] | 0 [0-0] | -47 [-80-14] |
| Variables are expressed as median [1^st^-3^rd^ quartile].  Not all outcome variables could be measured for each ΔPEEP level because of the fixed PEEP range explored in the decremental PEEP trial and the varying best PEEP value for each patient. Here follow the patients with missing ΔPEEP values:  ^1^Missing data for 16 patients (ID 1,2,3,6,7,8,11,12,13,14,15,16,17,18,19,20)  ^2^Missing data for 7 patients (ID 2,3,12,14,16,17,19)  ^3^Missing data for 2 patients (ID 3,17)  ^4^Missing data for 4 patients (ID 4,5,9,10)  ^5l^Missing data for 13 patients (ID 1,4,5,6,7,8,9,10,11,13,15,18,20)  ^6^Missing data for 18 patients (ID 1,2,4,5,6,7,8,9,10,11,12,13,14,15,16,18,19,20)  *Abbreviations*: EIT, electrical impedance tomography; PEEP, positive end-expiratory pressure; OD, lung overdistension; tot, total, i.e., referred to the entire lung; CL, lung collapse; GI, global inhomogeneity index; ΔEELI, difference of end-expiratory lung impedance compared to the value at 8 cmH_2_O of PEEP during phase 2. | | | |

| **Table E8: Effect of different phases and PEEP levels on EIT variables** | | | |
| --- | --- | --- | --- |
| OD_tot (%) | | | |
| PEEP ≥ best PEEP | | | |
| Variable | Coefficient | Standard error | P-value |
| Phase 2 | -7.18 | 0.49 | <0.001 |
| Phase 3 | -1.30 | 0.49 | 0.008 |
| Δ2 | 3.70 | 0.61 | <0.001 |
| Δ4 | 7.20 | 0.61 | <0.001 |
| Δ6 | 10.90 | 0.64 | <0.001 |
| Δ8 | 13.17 | 0.71 | <0.001 |
| Δ10 | 16.96 | 1.14 | <0.001 |
| PEEP < best PEEP | | | |
| Variable | Coefficient | Standard error | P-value |
| Phase 2 | -4.58 | 0.63 | <0.001 |
| Phase 3 | -1.36 | 0.63 | 0.033 |
| Δ-2 | 6.04 | 1.45 | <0.001 |
| Δ-4 | 3.28 | 1.45 | 0.026 |
| Δ-6 | 0.38 | 1.49 | 0.798 |
| CL_tot (%) | | | |
| PEEP ≥ best PEEP | | | |
| Variable | Coefficient | Standard error | P-value |
| Phase 2 | 10.64 | 0.65 | <0.001 |
| Phase 3 | 1.49 | 0.65 | 0.022 |
| Δ2 | -4.80 | 0.82 | <0.001 |
| Δ4 | -7.77 | 0.82 | <0.001 |
| Δ6 | -12.13 | 0.86 | <0.001 |
| Δ8 | -13.84 | 0.94 | <0.001 |
| Δ10 | -18.14 | 1.50 | <0.001 |
| PEEP < best PEEP | | | |
| Variable | Coefficient | Standard error | P-value |
| Phase 2 | 11.51 | 0.87 | <0.001 |
| Phase 3 | 1.36 | 0.87 | 0.122 |
| Δ-2 | -10.09 | 2.00 | <0.001 |
| Δ-4 | -6.29 | 2.00 | 0.002 |
| Δ-6 | -3.18 | 2.07 | 0.126 |
| GI_tot (%) | | | |
| PEEP ≥ best PEEP | | | |
| Variable | Coefficient | Standard error | P-value |
| Phase 2 | 8.22 | 0.85 | <0.001 |
| Phase 3 | 0.56 | 0.85 | 0.511 |
| Δ2 | -2.72 | 1.07 | 0.011 |
| Δ4 | -4.83 | 1.07 | <0.001 |
| Δ6 | -5.80 | 1.10 | <0.001 |
| Δ8 | -6.27 | 1.23 | <0.001 |
| Δ10 | -5.58 | 1.97 | 0.005 |
| PEEP < best PEEP | | | |
| Variable | Coefficient | Standard error | P-value |
| Phase 2 | 14.60 | 1.47 | <0.001 |
| Phase 3 | 1.11 | 1.47 | 0.452 |
| Δ-2 | -20.85 | 3.42 | <0.001 |
| Δ-4 | -16.77 | 3.42 | <0.001 |
| Δ-6 | -12.52 | 3.52 | <0.001 |
| ΔEELI_tot (%) | | | |
| PEEP ≥ best PEEP | | | |
| Variable | Coefficient | Standard error | P-value |
| Phase 2 | -202.51 | 9.81 | <0.001 |
| Phase 3 | -27.47 | 9.81 | 0.005 |
| Δ2 | 87.23 | 12.35 | <0.001 |
| Δ4 | 169.45 | 12.35 | <0.001 |
| Δ6 | 262.11 | 12.79 | <0.001 |
| Δ8 | 302.28 | 14.29 | <0.001 |
| Δ10 | 319.77 | 22.81 | <0.001 |
| PEEP < best PEEP | | | |
| Variable | Coefficient | Standard error | P-value |
| Phase 2 | -133.18 | 13.09 | <0.001 |
| Phase 3 | -34.44 | 13.09 | 0.010 |
| Δ-2 | 151.75 | 30.18 | <0.001 |
| Δ-4 | 89.90 | 30.22 | 0.004 |
| Δ-6 | 30.42 | 31.14 | 0.331 |
| ΔEELI ventral (%) | | | |
| PEEP ≥ best PEEP |  |  |  |
| Variable | Coefficient | Standard error | P-value |
| Phase 2 | -31.17 | 2.34 | <0.001 |
| Phase 3 | -3.69 | 2.34 | 0.12 |
| Δ2 | 16.28 | 2.94 | <0.001 |
| Δ4 | 29.13 | 2.94 | <0.001 |
| Δ6 | 42.87 | 3.05 | <0.001 |
| Δ8 | 51.61 | 3.40 | <0.001 |
| Δ10 | 62.6 | 10.32 | <0.001 |
| PEEP < best PEEP |  |  |  |
| Variable | Coefficient | Standard error | P-value |
| Phase 2 | -30.64 | 3.34 | <0.001 |
| Phase 3 | -6.02 | 3.34 | 0.07 |
| Δ-2 | 35.63 | 7.71 | <0.001 |
| Δ-4 | 20.49 | 7.72 | 0.01 |
| Δ-6 | 9.39 | 7.95 | 0.24 |
| ΔEELI mid-ventral (%) | | | |
| PEEP ≥ best PEEP |  |  |  |
| Variable | Coefficient | Standard error | P-value |
| Phase 2 | -68.32 | 5.52 | <0.001 |
| Phase 3 | -8.73 | 5.52 | 0.11 |
| Δ2 | 38.55 | 6.95 | <0.001 |
| Δ4 | 71.48 | 6.95 | <0.001 |
| Δ6 | 108.72 | 7.2 | <0.001 |
| Δ8 | 124.71 | 8.04 | <0.001 |
| Δ10 | 133.70 | 12.84 | <0.001 |
| PEEP < best PEEP |  |  |  |
| Variable | Coefficient | Standard error | P-value |
| Phase 2 | -50.56 | 6.88 | <0.001 |
| Phase 3 | -14.18 | 6.88 | 0.04 |
| Δ-2 | 63.05 | 15.89 | <0.001 |
| Δ-4 | 34.94 | 15.91 | 0.03 |
| Δ-6 | 4.51 | 16.38 | 0.78 |
| ΔEELI mid-dorsal (%) | | | |
| PEEP ≥ best PEEP |  |  |  |
| Variable | Coefficient | Standard error | P-value |
| Phase 2 | -72.24 | 3.8 | <0.001 |
| Phase 3 | -9.9 | 3.8 | 0.01 |
| Δ2 | 23.82 | 4.78 | <0.001 |
| Δ4 | 52.02 | 4.78 | <0.001 |
| Δ6 | 82.04 | 4.95 | <0.001 |
| Δ8 | 90.54 | 5.53 | <0.001 |
| Δ10 | 92.84 | 8.82 | <0.001 |
| PEEP < best PEEP |  |  |  |
| Variable | Coefficient | Standard error | P-value |
| Phase 2 | -37.44 | 4.11 | <0.001 |
| Phase 3 | -10.58 | 4.11 | 0.01 |
| Δ-2 | 49.88 | 9.46 | <0.001 |
| Δ-4 | 34.64 | 9.48 | <0.001 |
| Δ-6 | 14.74 | 9.77 | 0.13 |
| ΔEELI dorsal (%) | | | |
| PEEP ≥ best PEEP |  |  |  |
| Variable | Coefficient | Standard error | P-value |
| Phase 2 | -30.42 | 1.94 | <0.001 |
| Phase 3 | -5.22 | 1.94 | 0.01 |
| Δ2 | 8.4 | 2.44 | <0.001 |
| Δ4 | 16.55 | 2.44 | <0.001 |
| Δ6 | 28.09 | 2.53 | <0.001 |
| Δ8 | 32.78 | 2.83 | <0.001 |
| Δ10 | 36.66 | 4.51 | <0.001 |
| PEEP < best PEEP |  |  |  |
| Variable | Coefficient | Standard error | P-value |
| Phase 2 | -14.38 | 1.84 | <0.001 |
| Phase 3 | -3.6 | 1.84 | 0.05 |
| Δ-2 | 3.78 | 4.17 | 0.37 |
| Δ-4 | 0.17 | 4.19 | 0.87 |
| Δ-6 | 2.2 | 4.33 | 0.61 |
| The coefficients, standard errors, and p-values of the association between ΔPEEP, i.e., the difference between the set PEEP level and the EIT-based optimal PEEP, and each outcome variable are obtained through the application of mixed-effects models, including the individual patient as a random factor and the weight and ΔPEEP as fixed factors.  Reference for covariate “phase” is phase 1. Reference for covariate “ΔPEEP” is ΔPEEP 0 cmH_2_O for values ≥ best PEEP and ΔPEEP -8 cmH_2_O for values < best PEEP.  *Abbreviations:* EIT, electrical impedance tomography; PEEP, positive end-expiratory pressure; OD, lung overdistension; tot, total, i,e,, referred to the entire lung; CL, lung collapse; GI, global inhomogeneity index; ΔEELI, difference of end-expiratory lung impedance compared to the value at 8 cmH_2_O of PEEP during phase 2. | | | |

| **Table E9: Pairwise comparison of EIT variables between different ΔPEEP levels and study phases** | | | | | | | | | |
| --- | --- | --- | --- | --- | --- | --- | --- | --- | --- |
| OD_tot (%) | | | | | | | | | |
| *Comparison within study phase: Phase 1* | | | | | | | | | |
| PEEP ≥ best PEEP | | | | | PEEP < best PEEP | | | | |
| Δ2 | | | <0.001 | | Δ-2 | | | <0.001 | |
| Δ4 | | | <0.001 | | Δ-4 | | | 0.021 | |
| Δ6 | | | <0.001 | | Δ-6 | | | 0.838 | |
| Δ8 | | | <0.001 | |  | | |  | |
| Δ10 | | | <0.001 | |  | | |  | |
| *Comparison within study phase: Phase 2* | | | | | | | | | |
| PEEP ≥ best PEEP | | | | | PEEP < best PEEP | | | | |
| Δ2 | | | 0.001 | | Δ-2 | | | 0.184 | |
| Δ4 | | | <0.001 | | Δ-4 | | | 0.455 | |
| Δ6 | | | <0.001 | | Δ-6 | | | 0.753 | |
| Δ8 | | | <0.001 | |  | | |  | |
| Δ10 | | | <0.001 | |  | | |  | |
| *Comparison within study phase: Phase 3* | | | | | | | | | |
| PEEP ≥ best PEEP | | | | | PEEP < best PEEP | | | | |
| Δ2 | | | <0.001 | | Δ-2 | | | <0.001 | |
| Δ4 | | | <0.001 | | Δ-4 | | | <0.001 | |
| Δ6 | | | <0.001 | | Δ-6 | | | 0.212 | |
| Δ8 | | | <0.001 | |  | | |  | |
| Δ10 | | | <0.001 | |  | | |  | |
| *Comparison within PEEP level* | | | | | | | | | |
| Δ-8 | | | | Δ-6 | | | Δ-4 | | |
| Phase | 1 | | | Phase | | 1 | Phase | | 1 |
| 2 | 0.896 | | | 2 | | 0.075 | 2 | | <0.001 |
| 3 | 0.456 | | | 3 | | 0.554 | 3 | | 0.156 |
| Δ-2 | | | | Δ0 | | | Δ2 | | |
| Phase | 1 | | | Phase | | 1 | Phase | | 1 |
| 2 | <0.001 | | | 2 | | <0.001 | 2 | | <0.001 |
| 3 | 0.209 | | | 3 | | 0.079 | 3 | | 0.197 |
| Δ4 | | | | Δ6 | | | Δ8 | | |
| Phase | | 1 | | Phase | | 1 | Phase | | 1 |
| 2 | | <0.001 | | 2 | | <0.001 | 2 | | <0.001 |
| 3 | | 0.139 | | 3 | | 0.233 | 3 | | 0.346 |
| Δ10 | | | |  |  |  |  |  |  |
| Phase | | 1 | |  |  |  |  |  |  |
| 2 | | 0.132 | |  |  |  |  |  |  |
| 3 | | 0.549 | |  |  |  |  |  |  |
| CL_tot (%) | | | | | | | | | |
| *Comparison within study phase: Phase 1* | | | | | | | | | |
| PEEP ≥ best PEEP | | | | | PEEP < best PEEP | | | | |
| Δ2 | | | <0.001 | | Δ-2 | | | <0.001 | |
| Δ4 | | | <0.001 | | Δ-4 | | | <0.001 | |
| Δ6 | | | <0.001 | | Δ-6 | | | 0.018 | |
| Δ8 | | | <0.001 | |  | | |  | |
| Δ10 | | | <0.001 | |  | | |  | |
| *Comparison within study phase: Phase 2* | | | | | | | | | |
| PEEP ≥ best PEEP | | | | | PEEP < best PEEP | | | | |
| Δ2 | | | <0.001 | | Δ-2 | | | 0.009 | |
| Δ4 | | | <0.001 | | Δ-4 | | | 0.203 | |
| Δ6 | | | <0.001 | | Δ-6 | | | 0.352 | |
| Δ8 | | | <0.001 | |  | | |  | |
| Δ10 | | | <0.001 | |  | | |  | |
| *Comparison within study phase: Phase 3* | | | | | | | | | |
| PEEP ≥ best PEEP | | | | | PEEP < best PEEP | | | | |
| Δ2 | | | <0.001 | | Δ-2 | | | <0.001 | |
| Δ4 | | | <0.001 | | Δ-4 | | | <0.001 | |
| Δ6 | | | <0.001 | | Δ-6 | | | 0.035 | |
| Δ8 | | | <0.001 | |  | | |  | |
| Δ10 | | | <0.001 | |  | | |  | |
| *Comparison within PEEP level* | | | | | | | | | |
| Δ-8 | | | | Δ-6 | | | Δ-4 | | |
| Phase | 1 | | | Phase | | 1 | Phase | | 1 |
| 2 | 0.077 | | | 2 | | <0.001 | 2 | | <0.001 |
| 3 | 0.766 | | | 3 | | 0.354 | 3 | | 0.178 |
| Δ-2 | | | | Δ0 | | | Δ2 | | |
| Phase | 1 | | | Phase | | 1 | Phase | | 1 |
| 2 | <0.001 | | | 2 | | <0.001 | 2 | | <0.001 |
| 3 | 0.607 | | | 3 | | 0.141 | 3 | | 0.361 |
| Δ4 | | | | Δ6 | | | Δ8 | | |
| Phase | 1 | | | Phase | | 1 | Phase | | 1 |
| 2 | <0.001 | | | 2 | | <0.001 | 2 | | <0.001 |
| 3 | 0.189 | | | 3 | | 0.696 | 3 | | 0.478 |
| Δ10 | | | |  |  |  |  |  |  |
| Phase | 1 | | |  |  |  |  |  |  |
| 2 | 0.538 | | |  |  |  |  |  |  |
| 3 | 0.538 | | |  |  |  |  |  |  |
| GI_tot (%) | | | | | | | | | |
| *Comparison within study phase: Phase 1* | | | | | | | | | |
| PEEP ≥ best PEEP | | | | | PEEP < best PEEP | | | | |
| Δ2 | | | 0.279 | | Δ-2 | | | <0.001 | |
| Δ4 | | | 0.041 | | Δ-4 | | | <0.001 | |
| Δ6 | | | 0.013 | | Δ-6 | | | <0.001 | |
| Δ8 | | | 0.031 | |  | | |  | |
| Δ10 | | | 0.820 | |  | | |  | |
| *Comparison within study phase: Phase 2* | | | | | | | | | |
| PEEP ≥ best PEEP | | | | | PEEP < best PEEP | | | | |
| Δ2 | | | <0.001 | | Δ-2 | | | 0.006 | |
| Δ4 | | | <0.001 | | Δ-4 | | | 0.114 | |
| Δ6 | | | <0.001 | | Δ-6 | | | 0.150 | |
| Δ8 | | | <0.001 | |  | | |  | |
| Δ10 | | | <0.001 | |  | | |  | |
| *Comparison within study phase: Phase 3* | | | | | | | | | |
| PEEP ≥ best PEEP | | | | | PEEP < best PEEP | | | | |
| Δ2 | | | 0.051 | | Δ-2 | | | <0.001 | |
| Δ4 | | | <0.001 | | Δ-4 | | | <0.001 | |
| Δ6 | | | 0.002 | | Δ-6 | | | <0.001 | |
| Δ8 | | | <0.001 | |  | | |  | |
| Δ10 | | | 0.102 | |  | | |  | |
| *Comparison within PEEP level* | | | | | | | | | |
| Δ-8 | | | | Δ-6 | | | Δ-4 | | |
| Phase | | 1 | | Phase | | 1 | Phase | | 1 |
| 2 | | 0.056 | | 2 | | <0.001 | 2 | | <0.001 |
| 3 | | 0.350 | | 3 | | 0.762 | 3 | | <0.001 |
| Δ-2 | | | | Δ0 | | | Δ2 | | |
| Phase | | 1 | | Phase | | 1 | Phase | | 1 |
| 2 | | <0.001 | | 2 | | <0.001 | 2 | | <0.001 |
| 3 | | 0.633 | | 3 | | 0.332 | 3 | | 0.71 |
| Δ4 | | | | Δ6 | | | Δ8 | | |
| Phase | | 1 | | Phase | | 1 | Phase | | 1 |
| 2 | | <0.001 | | 2 | | <0.001 | 2 | | 0.156 |
| 3 | | 0.982 | | 3 | | 0.599 | 3 | | 0.786 |
| Δ10 | | | |  |  |  |  |  |  |
| Phase | | 1 | |  |  |  |  |  |  |
| 2 | | 0.918 | |  |  |  |  |  |  |
| 3 | | 0.545 | |  |  |  |  |  |  |
| ΔEELI_tot (%) | | | | | | | | | |
| *Comparison within study phase: Phase 1* | | | | | | | | | |
| PEEP ≥ best PEEP | | | | | PEEP < best PEEP | | | | |
| Δ2 | | | <0.001 | | Δ-2 | | | <0.001 | |
| Δ4 | | | <0.001 | | Δ-4 | | | 0.003 | |
| Δ6 | | | <0.001 | | Δ-6 | | | 0.301 | |
| Δ8 | | | <0.001 | |  | | |  | |
| Δ10 | | | <0.001 | |  | | |  | |
| *Comparison within study phase: Phase 2* | | | | | | | | | |
| PEEP ≥ best PEEP | | | | | PEEP < best PEEP | | | | |
| Δ2 | | | <0.001 | | Δ-2 | | | 0.012 | |
| Δ4 | | | <0.001 | | Δ-4 | | | 0.138 | |
| Δ6 | | | <0.001 | | Δ-6 | | | 0.583 | |
| Δ8 | | | <0.001 | |  | | |  | |
| Δ10 | | | <0.001 | |  | | |  | |
| *Comparison within study phase: Phase 3* | | | | | | | | | |
| PEEP ≥ best PEEP | | | | | PEEP < best PEEP | | | | |
| Δ2 | | | <0.001 | | Δ-2 | | | <0.001 | |
| Δ4 | | | <0.001 | | Δ-4 | | | <0.001 | |
| Δ6 | | | <0.001 | | Δ-6 | | | 0.266 | |
| Δ8 | | | <0.001 | |  | | |  | |
| Δ10 | | | <0.001 | |  | | |  | |
| *Comparison within PEEP level* | | | | | | | | | |
| Δ-8 | | | | Δ-6 | | | Δ-4 | | |
| Phase | | 1 | | Phase | | 1 | Phase | | 1 |
| 2 | | 0.589 | | 2 | | 0.022 | 2 | | <0.001 |
| 3 | | 0.839 | | 3 | | 0.397 | 3 | | 0.112 |
| Δ-2 | | | | Δ0 | | | Δ2 | | |
| Phase | | 1 | | Phase | | 1 | Phase | | 1 |
| 2 | | <0.001 | | 2 | | <0.001 | 2 | | <0.001 |
| 3 | | 0.054 | | 3 | | 0.050 | 3 | | 0.037 |
| Δ4 | | | | Δ6 | | | Δ8 | | |
| Phase | | 1 | | Phase | | 1 | Phase | | 1 |
| 2 | | <0.001 | | 2 | | <0.001 | 2 | | <0.001 |
| 3 | | 0.499 | | 3 | | 0.291 | 3 | | 0.815 |
| Δ10 | | | |  |  |  |  |  |  |
| Phase | | 1 | |  |  |  |  |  |  |
| 2 | | 0.004 | |  |  |  |  |  |  |
| 3 | | 0.948 | |  |  |  |  |  |  |
| Linear mixed-effects models were conducted for each ΔPEEP level. i.e., the difference between the set PEEP level and the EIT-based optimal PEEP, and study phase for pairwise comparisons relative to the effect of weight application and PEEP, respectively. In the comparison within phase reference for PEEP ≥ best PEEP is ΔPEEP=0; reference for PEEP > best PEEP is ΔPEEP = -8.  *Abbreviations*: EIT, electrical impedance tomography; PEEP, positive end-expiratory pressure; OD, lung overdistension; tot, total. i.e., referred to the entire lung; CL, lung collapse; GI, global inhomogeneity index; ΔEELI, difference of end-expiratory lung impedance compared to the value at 8 cmH_2_O of PEEP during phase 2. | | | | | | | | | |
